# Early Changes in Exo- and Endocytosis in the EAE Mouse Model of Multiple Sclerosis Correlate with Decreased Synaptic Ribbon Size and Reduced Ribbon-Associated Vesicle Pools in Rod Photoreceptor Synapses

**DOI:** 10.3390/ijms221910789

**Published:** 2021-10-06

**Authors:** Ajay Kesharwani, Karin Schwarz, Ekta Dembla, Mayur Dembla, Frank Schmitz

**Affiliations:** 1 Department of Neuroanatomy, Institute of Anatomy and Cell Biology, Medical School, Saarland University, 66421 Homburg, Germany; karin.schwarz@uks.eu (K.S.); aku21085@gmail.com (E.D.); dotdot.mayur@gmail.com (M.D.); frank.schmitz@uks.eu (F.S.); 2 Department of Ophthalmology, Duke University School of Medicine, Durham, NC 27710, USA

**Keywords:** multiple sclerosis, optic neuritis, EAE mouse model, rod photoreceptor, ribbon synapses, synaptic ribbon, exocytosis, endocytosis, dynamin, phospho-dynamin

## Abstract

Multiple sclerosis (MS) is an inflammatory disease of the central nervous system that finally leads to demyelination. Demyelinating optic neuritis is a frequent symptom in MS. Recent studies also revealed synapse dysfunctions in MS patients and MS mouse models. We previously reported alterations of photoreceptor ribbon synapses in the experimental auto-immune encephalomyelitis (EAE) mouse model of MS. In the present study, we found that the previously observed decreased imunosignals of photoreceptor ribbons in early EAE resulted from a decrease in synaptic ribbon size, whereas the number/density of ribbons in photoreceptor synapses remained unchanged. Smaller photoreceptor ribbons are associated with fewer docked and ribbon-associated vesicles. At a functional level, depolarization-evoked exocytosis as monitored by optical recording was diminished even as early as on day 7 after EAE induction. Moreover compensatory, post-depolarization endocytosis was decreased. Decreased post-depolarization endocytosis in early EAE correlated with diminished synaptic enrichment of dynamin3. In contrast, basal endocytosis in photoreceptor synapses of resting non-depolarized retinal slices was increased in early EAE. Increased basal endocytosis correlated with increased de-phosphorylation of dynamin1. Thus, multiple endocytic pathways in photoreceptor synapse are differentially affected in early EAE and likely contribute to the observed synapse pathology in early EAE.

## 1. Introduction

Multiple sclerosis (MS) is a chronic inflammatory disease of the central nervous system (CNS) leading to axonal demyelination and glial scars in the white matter [1,2,3,4]. The pathogenesis of MS is not completely understood. Recent studies on MS patients [5,6,7,8,9,10,11] and mouse models of MS [12,13,14,15] also demonstrated changes in the grey matter of the CNS. Remarkably, some grey matter alterations occurred before obvious changes in the white matter, arguing that the grey matter changes are not just secondary to axonal demyelination [7,8,15,16,17]. Alterations in the grey matter include neuronal cell death and synapse dysfunction [13,15,18,19].

The visual system is often affected in MS. Optic neuritis is a frequent early symptom in MS patients [20] and in animal models of MS, including the widely accepted EAE mouse model of MS [21,22,23]. The retina is an unmyelinated tissue. Of note, retinal synapses were morphologically and functionally compromised in the experimental auto-immune encephalomyelitis (EAE) mouse model of MS [24,25]. Synapse alterations occurred in the pre-clinical phase even before the onset of optic nerve demyelination both in MS patients as well as in the EAE mouse model [7,24,25]. In the retina, photoreceptor synapses were particularly strongly affected by EAE [24,25]. Photoreceptor synapses are continuously active ribbon synapses that contain large presynaptic specializations, the synaptic ribbons [26,27,28]. The membrane-proximal base of the ribbon is anchored to the active zone close to the presynaptic Cav-channels that are crucial to trigger exocytosis [28]. Synaptic ribbons are considered as specializations needed for maintaining continuous presynaptic vesicle trafficking [28]. The RIBEYE protein is a unique and main component of synaptic ribbons [29,30].

In a previous study [24], we showed that RIBEYE immunosignals were decreased in rod photoreceptor synapses of EAE mice at an early pre-clinical stage. Rod synapses represent the major photoreceptor synapse population in the mouse retina (≈95% of photoreceptor synapses) [28]. In contrast to cone synapses, rod synapses possess only a single active zone with a single particularly large synaptic ribbon. In the present study, we sought to determine the reason for the decreased RIBEYE immunosignals in rod photoreceptor ribbon synapses of EAE mice at the early pre-clinical stage. We found that the number of rod photoreceptor synaptic ribbons is unchanged in early EAE but that rod synaptic ribbons are smaller in size. The decrease in synaptic ribbon size in rod synapses is accompanied not only by a decrease in depolarization-evoked synaptic vesicle exocytosis (as early as on day 7 after injection) but also by a decrease in post-stimulus vesicle endocytosis. These data emphasize the role of the synaptic ribbon for depolarization-evoked exocytic and endocytic vesicle trafficking in early EAE. In contrast to the decreased evoked responses, basal vesicle cycling under resting conditions was elevated in early EAE photoreceptors, most likely resulting from elevated basal presynaptic Ca^2+^ levels that were recently identified [25].

## 2. Results

**Figure 1:**
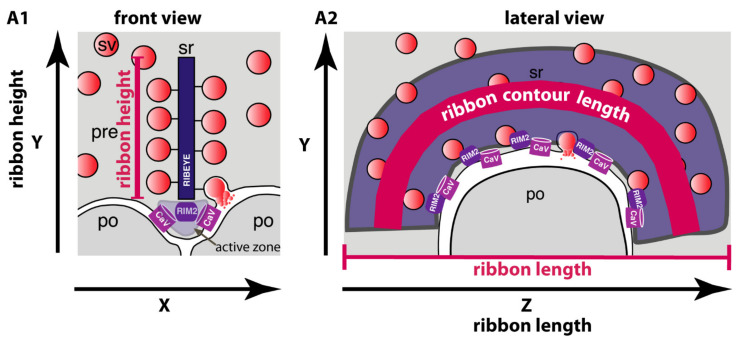
Schematic drawing of a rod photoreceptor synapse in two different views. (**A1**) Cross section of a rod photoreceptor synapse showing a bar-shaped synaptic ribbon that is anchored perpendicularly to the active zone. Rod photoreceptor ribbon synapse sectioned in this way were considered for the measurement of the height of synaptic ribbon. (**A2**) Lateral view of horseshoe-shaped synaptic ribbon that runs in parallel to the active zone. For the measurement of the synaptic ribbon length (in z-direction), serial ultrathin sections were analyzed by transmission electron microscopy. Abbreviations: po, post-synaptic; pre, pre-synaptic, sr, synaptic ribbon; sv, synaptic vesicle.

Synaptic ribbons are particularly large in rod photoreceptor synapses and display a typical complex three-dimensional morphology [28,31] (see also Figure 1). In a previous study [24], we found that the RIBEYE immunosignals of rod photoreceptor synaptic ribbons in the OPL were significantly weaker in early EAE mice in comparison to photoreceptor synaptic ribbons from the CFA-injected control group (at 9 days after injection). In the present study, we asked for the reasons of the decreased RIBEYE immunosignals of photoreceptor synaptic ribbons in early EAE mice.

**Figure 2:**
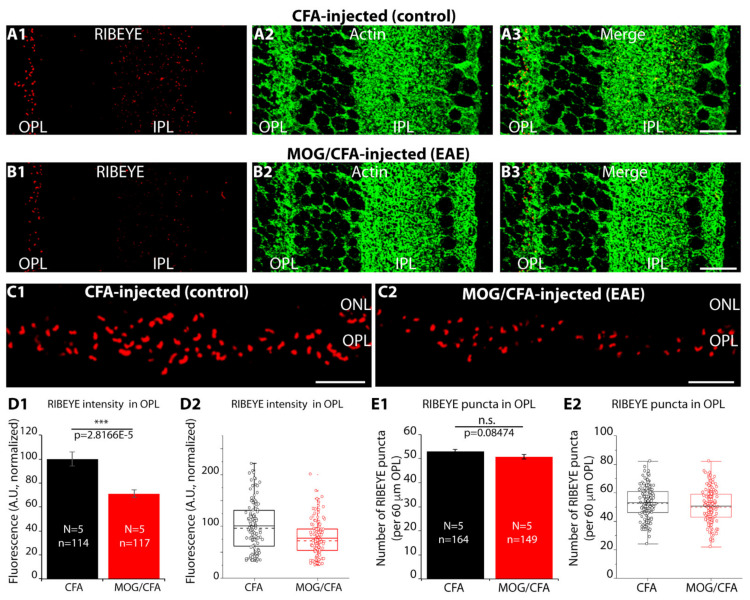
The number of RIBEYE immunolabelled puncta in the OPL is unchanged but decreased in intensity in early pre-clinical EAE. (**A1**–**C2**) 0.5 µm semi-thin retina sections from CFA (control) and MOG/CFA immunized C57BL/6J mice were processed for immunolabeling on day 9 after the injection. Sections were double-immunolabelled with rabbit polyclonal antibody against RIBEYE (U2656) and mouse monoclonal antibody against actin (Clone C4), as indicated in Figure (**A1**–**C2**). The individual immunosignals from A1 and A2/B1 and B2 were superimposed in A3/B3. (**C1**,**C2**) Magnified view of the OPL immuno-labelled with anti-RIBEYE antibody to visualize the synaptic ribbons. (**D1**,**D2**) Quantification of fluorescence intensity (integrated density) of RIBEYE immunosignals in the OPL. (**E1**,**E2**) Quantification of the number of RIBEYE puncta in the OPL. Values in (**D1**,**E1**) are means ± S.E.M. In the box-and-whiskers plot (**D2**,**E2**) of the data from (**D1**,**E1**), mean values are indicated by solid horizontal lines; median values by dotted horizontal lines. Boxes represent the 25th–75th percentiles of data points and whiskers are equal to 1.5 times of the interquartile range (IQR). Student’s-*t*-test was applied to determine the statistical significance for puncta count (**E1**) and Mann–Whitney U-test for fluorescence intensity (**D1**). Abbreviations: OPL, outer plexiform layer; IPL, inner plexiform layer; ONL, outer nuclear layer; S.E.M., standard error of the mean; N, number of mice; n, number of confocal images analyzed to quantify fluorescence intensity and to count the puncta numbers from retina sections; ***, *p* ≤ 0.001; n.s., not significant. Scale bars: 10 µm (**A**,**B**); 5 µm (**C**).

First, we re-analyzed the intensity of RIBEYE immunosignals in the OPL of retinas from MOG/CFA- and CFA-immunized mice isolated on day 9 after injection and observed a similar reduction in RIBEYE immunolabelling intensity in MOG/CFA-injected mice in comparison to CFA-injected control mice (Figure 2(A1–C2); for quantification, see Figure 2(D1,D2)), as previously published [24]. Anti-actin immunostaining served as a reference in these experiments, similarly as previously described [24]. Next, we counted the number of RIBEYE/ribbon puncta per 60 µm of OPL. Remarkably, the number of RIBEYE puncta per 60 µm of OPL (ribbon density) was unchanged between MOG/CFA-injected mice and CFA-injected control mice (Figure 2(E1,E2)). Thus, the number of ribbons in photoreceptor synapses does not differ between EAE and the control group and cannot be the reason for the decreased RIBEYE immunosignals in photoreceptor synapses of early EAE mice. Thus, we tested whether individual rod synaptic ribbons differ between MOG/CFA-injected early EAE mice and control mice on day 9 after injection.

### 2.1. Photoreceptor Ribbons Are Shorter in Early EAE Mice in Comparison to Control Mice as Judged by 3D SR-SIM Analyses

**Figure 3:**
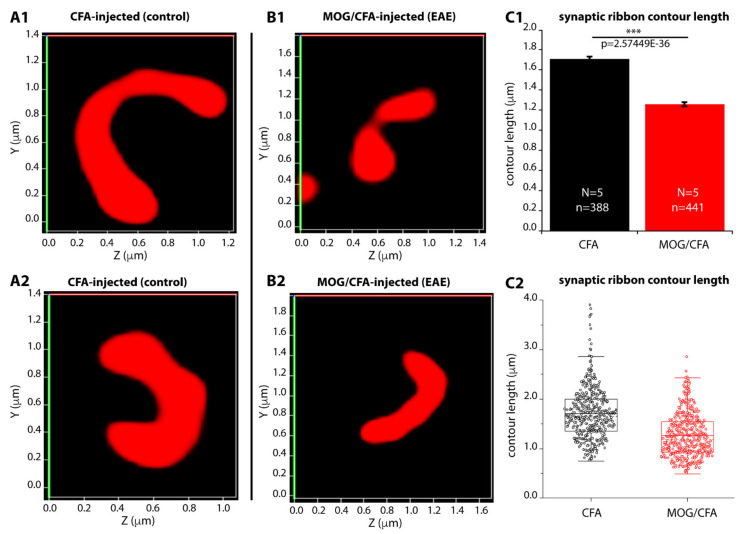
Contour length of rod photoreceptor synaptic ribbon is reduced in early pre-clinical EAE mice in comparison to control mice. (**A1**–**B2**) Representative 3D SR-SIM images of individual synaptic ribbons from rod photoreceptor synapses of CFA-injected control mice (**A1**,**A2**) and MOG/CFA-injected EAE mice (**B1**,**B2**) immunolabelled with mouse monoclonal antibody against RIBEYE (2D9). (**C1**,**C2**) Quantitative analyses of the contour lengths of rod photoreceptor synaptic ribbons measured as maximum 2D projections from the 3D SR-SIM images (see also Figure 1). Values in (**C1**) are means ± S.E.M. (1.71 ± 0.02 µm in CFA-injected control mice; 1.26 ± 0.02 µm in MOG/CFA-injected EAE mice). In the box-and-whiskers plot (**C2**) of the data from (**C1**), mean values are indicated by solid horizontal lines; median values by dotted horizontal lines. Boxes represent the 25th–75th percentiles of data points and whiskers are equal to 1.5 times of the interquartile range (IQR). Mann–Whitney U-test was used to determine the statistical significance. N, number of mice; n, number of analyzed 3D SR-SIM images; ***, *p* ≤ 0.001.

For this purpose, we analyzed the length of individual synaptic ribbons of rod photoreceptor synapses in early EAE mice (vs. the control group) by 3D super-resolution structured illumination microscopy (3D SR-SIM) (Figure 3). 3D SR-SIM is particularly useful to examine the contour length (Figure 1(A2)) of synaptic ribbons [24,25]. Using 3D SR-SIM, we found that the contour length of photoreceptor synaptic ribbons was significantly smaller in MOG/CFA-injected mice in comparison to CFA-injected control mice on day 9 after injection (Figure 3(A1–B2); for quantification, Figure 3(C1,C2)). Thus, a decreased photoreceptor ribbon length appears to account for the decreased RIBEYE immunosignals of photoreceptor synaptic ribbons in early EAE.

### 2.2. Synaptic Ribbons Are Shorter in Height and Length in Early EAE Mice in Comparison to Litermate Control Mice as Judged by Transmission Electron Microscopy

**Figure 4:**
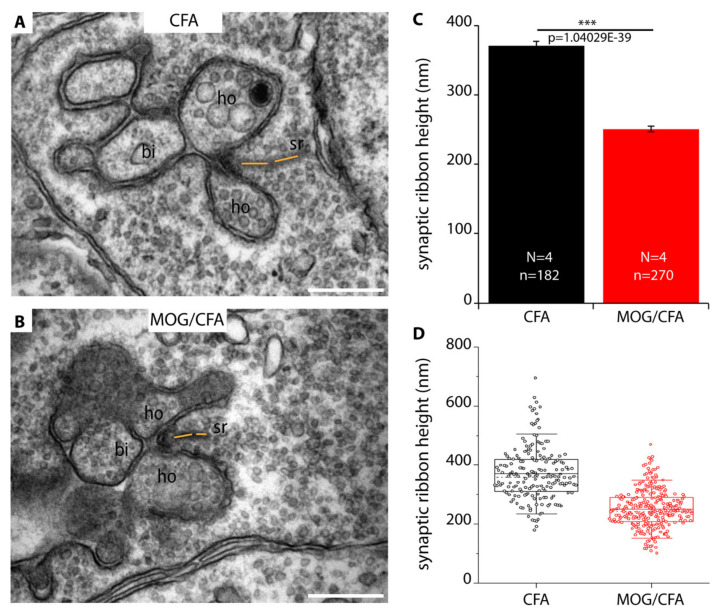
The height of rod photoreceptor synaptic ribbon is shorter in early pre-clinical EAE mice. (**A**,**B**) Representative transmission EM images of cross-sectioned synaptic ribbon from rod-photoreceptor synapses of CFA-injected control mice (**A**) and MOG/CFA-injected EAE mice (**B**) obtained on day 9 after injection. The dashed orange line in (**A**,**B**) demonstrate how synaptic ribbon height was determined. Values in (**C**) are means ± S.E.M (370 nm ± 7 nm ribbon height in CFA-injected control mice; 251 nm ± 4 nm in MOG/CFA-injected EAE mice). In the box-and-whiskers plot (**D**) of the data from (**C**), mean values are indicated by solid horizontal lines; median values by dotted horizontal lines. Boxes represent the 25th–75th percentiles of data points and whiskers are equal to 1.5 times of the interquartile range (IQR). Mann–Whitney U-test was used to determine the statistical significance. Abbreviations: sr, synaptic ribbon; ho, horizontal cell dendrite; bi, bipolar cell dendrite; N, number of mice; n, number of electron microscope images of rod-photoreceptor ribbon synapses analyzed; ***, *p* ≤ 0.001. Scale bars: 500 nm (**A**,**B**).

**Figure 5:**
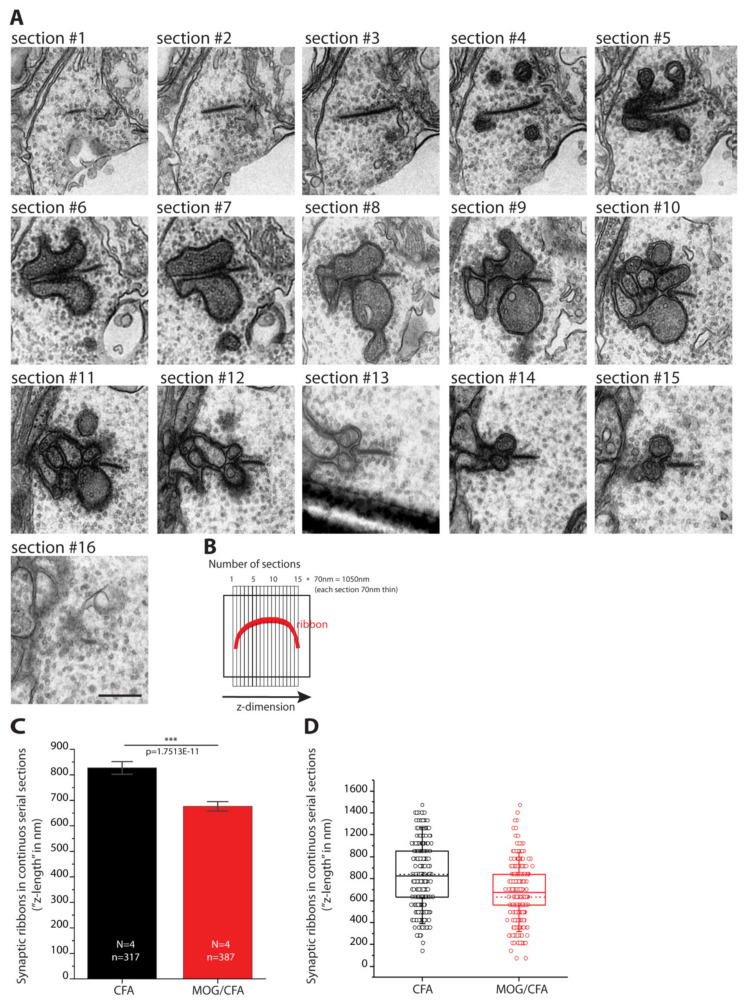
Synaptic ribbon length (“z-length”) in rod photoreceptor synapses of early pre-clinical EAE mice is shorter than in control littermate mice, as judged by transmission electron microscopy of serial ultrathin sections. (**A**) Representative electron microscopic images of a single photoreceptor ribbon synapse acquired from serial ultrathin retina sections of a CFA-injected control mouse obtained 9 days after injection. (**B**) schematically demonstrates how the z-length of rod synaptic ribbons was determined by counting the number of ultrathin sections in which an individual synaptic ribbon is present. Values in (**C**) are means ± S.E.M. of the synaptic ribbon length in z-direction (827 nm ± 16 nm in CFA-injected control mice and 677 nm ± 12 nm in MOG/CFA-injected EAE mice). In the box-and-whiskers plot (**D**) of the data from (**C**), mean values are indicated by solid horizontal lines; median values by dotted horizontal lines. Boxes represent the 25th–75th percentiles of data points and whiskers are equal to 1.5 times of the interquartile range (IQR). Mann–Whitney U-test was used to determine the statistical significance. N, number of mice; n, number of ribbon synapses analyzed to measure the z-axis length; ***, *p* ≤ 0.001. Scale bars: 500 nm (scale bar in section #16 applies to all sections).

In order to further characterize and confirm these light microscopical alterations of synaptic ribbon size in early EAE photoreceptor synapses, we performed ultrastructural analyses by using transmission electron microscopy (TEM). First, we determined the height of synaptic ribbons in cross-sections of rod photoreceptor synapses of MOG/CFA-injected EAE mice and in CFA-injected control mice obtained on day 9 after injection (Figure 4). For this purpose, the height of cross-sectioned ribbons was measured from its anchorage site in the active zone to the free cytosolic end in rod photoreceptor synapses (see also Figure 1(A1)). Only rod photoreceptor synaptic ribbons were included in these analyses that were anchored to a clearly visible active zone (presynaptic density, arciform density) and were opposed by clearly visible postsynaptic triads. By this procedure, we wanted to exclude that tangentially sectioned ribbons (sectioned parallel to the active zone, representing ribbon “length” instead of ribbon “height”; Figure 1(A1,A2)) were erroneously included in these analyses. We found that the ribbon height in rod photoreceptor synapses was significantly lower in MOG/CFA-injected mice than in CFA-injected control mice (Figure 4A,B; for quantification, Figure 4C,D). Similarly, also the extension of rod ribbons in z-direction (ribbon length, Figure 1(A2)), as determined by the number of serial ultrathin EM sections in which a single synaptic ribbon could be unambiguously observed, was smaller in MOG/CFA-injected mice in comparison to CFA-injected control mice (Figure 5). Estimating a section thickness of 70 nm per ultrathin EM section, we determined that a rod ribbon extends to about 677 nm ± 12 nm in z-direction in MOG/CFA-injected early EAE mice and about 827 nm ± 16 nm in CFA-injected control mice (Figure 5C,D). Please note that the z-length of synaptic ribbons as measured by counting the number of serial EM sections, in which an individual ribbon appeared, is smaller than the contour length of the synaptic ribbons (Figure 1(A2)) because counting of ribbon-containing serial sections does not take into account that ribbons are bended in 3D and have a horseshoe-shaped appearance (Figure 1(A2)). The z-length as measured by the number of ultrathin sections in which a ribbon is present mostly corresponds to the 2D projection of the 3D ribbon into a single plane (Figure 5B) and the actual length is underestimated by this approach. In conclusion, the ultrastructural analyses confirm the results of the 3D SR-SIM analyses in which shorter synaptic ribbons were observed in rod photoreceptor synapses of early EAE mice (Figure 3).

### 2.3. The Number of Docked Vesicles and Ribbon-Tethered Vesicles Are Reduced in Early EAE Mice in Comparison to Control Mice

**Figure 6:**
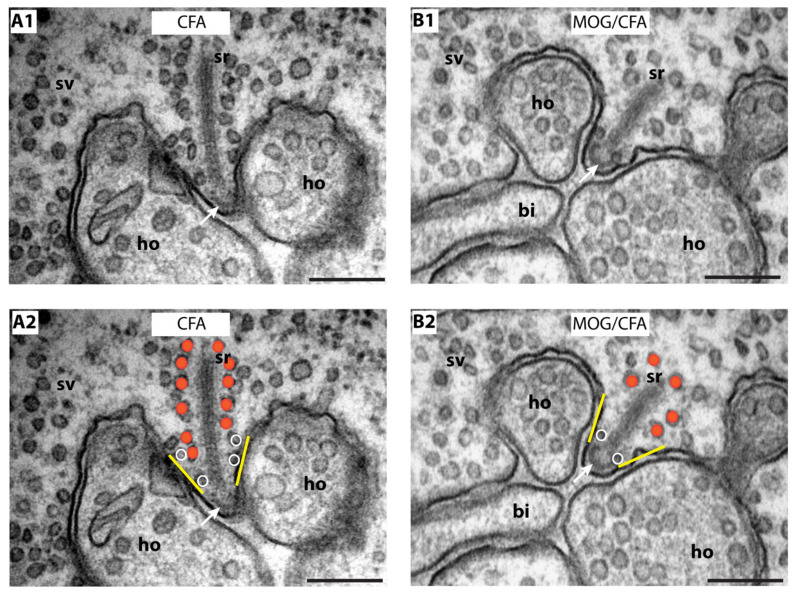

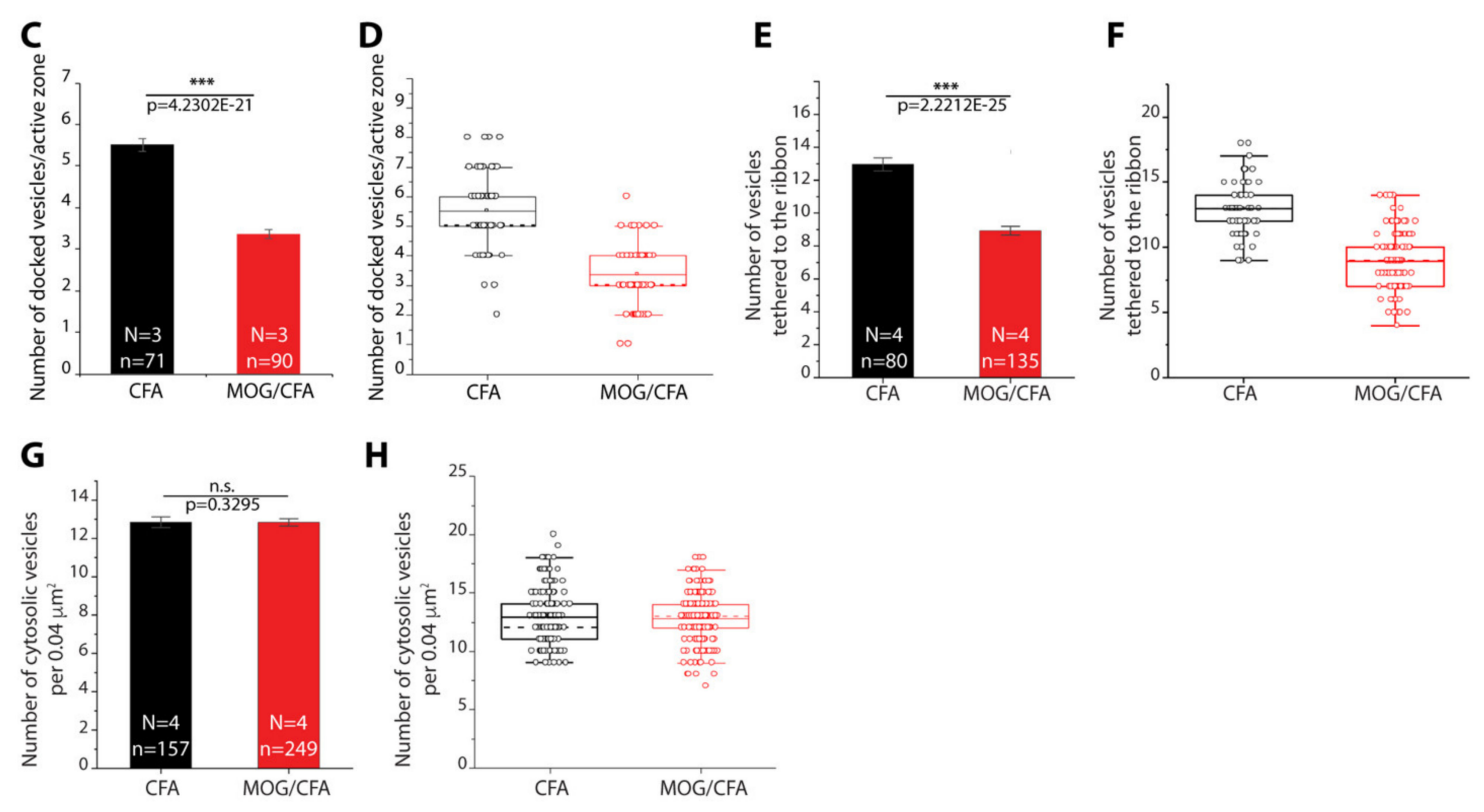
The number of docked vesicles and ribbon-tethered vesicles are reduced in early EAE mice in comparison to control mice. (**A1**–**B2**) Representative TEM images of rod photoreceptor synapses from CFA-injected control mice (**A1**,**A2**) and MOG/CFA-injected EAE mice (**B1**,**B2**). The yellow lines in (**A2**,**B2**) denote the 150 nm range in vicinity of the active zone in which the docked vesicles (white circles) were counted. The red spheres in (**A2**,**B2**) exemplify the ribbon-tethered synaptic vesicles. White arrows in (**A1**–**B2**) point to the arciform density. (**C**,**D**) Average number of docked vesicles (CFA: 5.5 ± 0.14 vesicles; MOG/CFA: 3.1 ± 0.10 vesicles). (**E**,**F**) Average number of ribbon tethered vesicles (CFA: 12.96 ± 0.26 vesicles; MOG/CFA: 8.93 ± 0.17 vesicles). (**G**,**H**) Average number of cytosolic vesicles determined in a square area of 200 × 200 nm within the presynaptic terminal in distance from the synaptic ribbon (CFA: 12.85 ± 0.18 vesicles; MOG/CFA: 12.83 ± 0.12 vesicles). Values in (**C**,**E**,**G**) are means ± S.E.M. In the box-and-whiskers plots (**D**,**F**,**H**) of the data from (**C**,**E**,**G**), mean values are indicated by solid horizontal lines; median values by dotted horizontal lines. Boxes represent the 25th–75th percentiles of data points and whiskers are equal to 1.5 times of the interquartile range (IQR). Mann–Whitney U-test was used to determine the statistical significance. Abbreviations: ho, horizontal cell dendrite; bi, bipolar cell dendrite; sv, synaptic vesicles; sr, synaptic ribbon; N, number of mice; n, number of electron microscope images of rod-photoreceptor ribbon synapses analyzed to count the synaptic vesicles; ***, *p* ≤ 0.001; n.s., not significant. Scale bars: 200 nm.

Next, we counted docked synaptic vesicles, ribbon-tethered, and reserve pool synaptic vesicles in the center of the presynaptic terminal, as previously described [30]. We determined the size of these vesicle pools because they are important determinants of synaptic transmission [28,31,32]. We found that both ribbon-tethered vesicles as well as vesicles docked to the active zone were decreased in number in MOG/CFA-injected mice in comparison to CFA-injected control mice (Figure 6A,B; for quantification, Figure 6C–F). In contrast the number of synaptic vesicles in the cytosol (reserve pool) of rod photoreceptor synapses was found to be unchanged between MOG/CFA-injected EAE mice and CFA-injected littermate control mice (Figure 6G,H).

As outlined in the introduction, the synaptic ribbon has a well-established role in synaptic vesicle exocytosis (for review, [26,28,32]). The synaptic ribbon is affected by mutations of endocytic proteins [33,34,35] and might thus also play a role in endocytic vesicle retrieval (e.g., [28,33,34,35,36,37,38,39,40,41,42]). Therefore, we analyzed in detail whether the alterations of synaptic ribbons are associated with changes in evoked and basal synaptic vesicle cycling.

### 2.4. Defects in Depolarization-Evoked Exocytosis in EAE Mice as Early as on Day 7 after Injection

**Figure 7:**
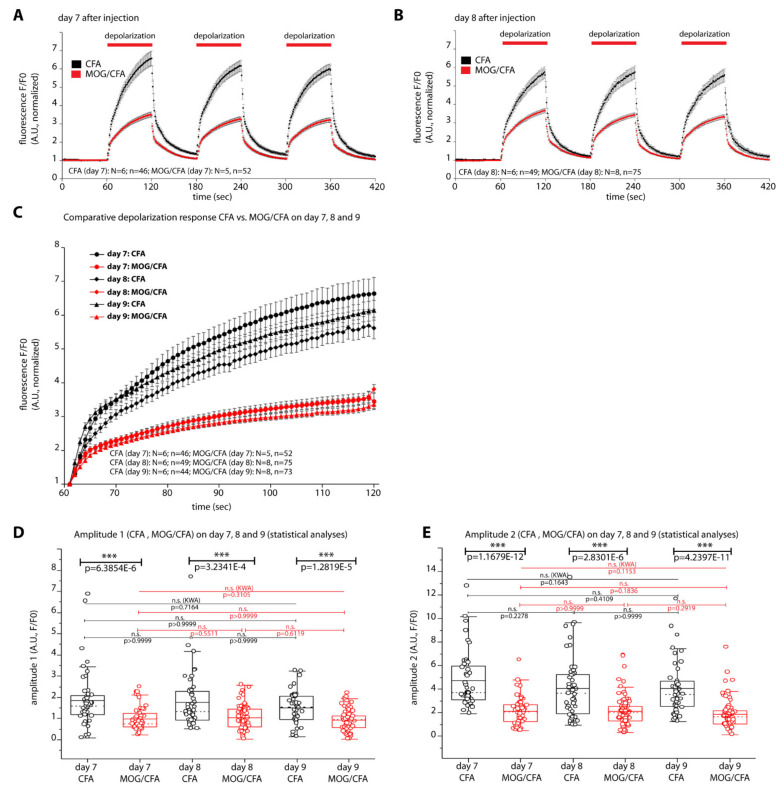
Synaptic vesicle exocytosis in EAE mice is compromised as early as on day 7 after injection. Exocytosis was analyzed by optical recording of SypHy responses, as described (Dembla et al., 2018) at the indicated days after injection. (**A**,**B**) K^+^-depolarization-evoked SypHy responses in the OPL of MOG/CFA-injected EAE mice and CFA-injected control mice on day 7 and day 8 after injection. (**C**) Comparison of depolarization-evoked responses between CFA-injected control mice group and MOG-CFA-injected EAE mice group on day 7, day 8, and day 9 after injection. All data could be best fitted by double-exponential curves from which fast and slow depolarization-evoked responses (amplitude 1 and amplitude 2) were extracted (amplitudes plotted in (**D**,**E**)). (**D**,**E**) Comparison of amplitude 1 and amplitude 2 from CFA-injected control vs. MOG/CFA-injected EAE mice on day 7, day 8, and day 9 after injection. Mann–Whitney U-tests were applied for comparing separately amplitude 1 (**D**) and amplitude 2 (**E**) between CFA-injected (control) and MOG/CFA-injected EAE mice on day 7, day 8, and day 9 after injection (bold black horizontal lines in (**D**,**E**)). Comparison of amplitudes of fast and slow depolarization evoked responses (amplitude 1 and amplitude 2) between day 7, day 8, and day 9 after injection by Kruskal–Wallis ANOVA (KWA) test in MOG/CFA-injected EAE mice (thin red horizontal lines) and CFA-injected control mice (thin black horizontal lines) with post-hoc Dunn analyses for multiple comparisons. In the box-and-whiskers plots (**D**,**E**), mean values are indicated by solid horizontal lines; median values by dotted horizontal lines. Boxes represent the 25th–75th percentiles of data points and whiskers are equal to 1.5 times of the interquartile range (IQR). KWA, Kruskal–Wallis ANOVA; N, number of mice; n, number of retinal slices used for optical recording; ***, *p* ≤ 0.001; n.s., not significant.

A previous study already showed defects in depolarization-evoked synaptic vesicle exocytosis on day 9 after injection [24]. On that same time point (9 days after injection) we performed the described morphological analyses. Thus, the published functional changes observed on day 9 after injection [24] correlate with the morphological changes described in the present study. Since a recent study [25] found alterations of presynaptic Ca^2+^ signaling even at earlier time points, i.e., on day 7 and day 8 after injection, we also analyzed mice obtained on day 8 and day 7 after injection for possible defects in synaptic vesicle exocytosis. For this purpose, we monitored synaptic vesicle exocytosis by optical recording of SypHy fluorescence from our transgenic SypHy reporter mice, exactly as previously described [24], but now on retinal slices obtained from mice on day 8 and day 7 after injection of MOG/CFA (EAE group) or CFA (control group) (Figure 7). Indeed, we found on these even earlier time points (day 7, day 8 post injection) significantly reduced high K^+^-depolarization—evoked responses of synaptic vesicle exocytosis in the OPL of MOG/CFA-injected mice in comparison to CFA-injected control mice (Figure 7A,B). The depolarization-evoked responses could be best fitted with a double-exponential fit both in MOG/CFA- injected mice as well as in CFA-injected control mice (Figure 7C). The amplitudes of the fast and slow depolarization-evoked responses (amplitude 1 and amplitude 2) were calculated from these curve fits (Figure 7D,E). We found on all days analyzed (day 7, day 8, and day 9 after injection) significantly reduced values for amplitude 1 and amplitude 2 in the OPL of MOG/CFA-injected mice in comparison to CFA-injected control mice (Figure 7D,E). Similar to the previously described alterations in presynaptic Ca^2+^ signaling [25], the reduction of synaptic vesicle exocytosis was already maximally reduced on day 7 after injection and did not differ significantly on day 7 from the values observed on day 8 and day 9 after injection (Figure 7D,E).

### 2.5. Post-Stimulus Endocytosis Is Decreased in Early EAE Mice in Comparison to Control MICE

**Figure 8:**
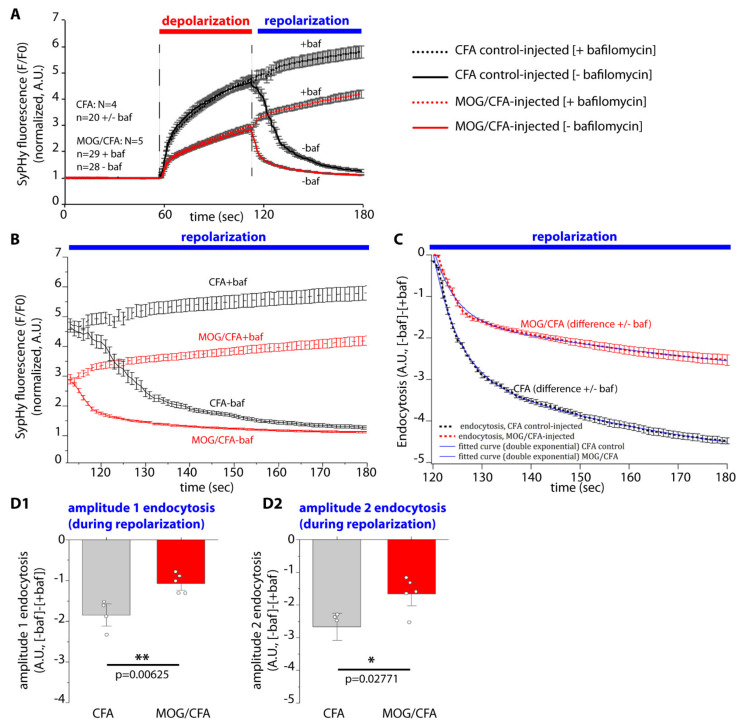
Post-stimulus endocytosis is decreased in MOG/CFA-injected early EAE mice in comparison to CFA-injected control mice. (**A**) Optical recording of SypHy response measurement in the presence and absence of bafilomycin A1 in the OPL of MOG/CFA-injected EAE mice and CFA-injected control mice on day 9 after injection. (**B**) SypHy responses in the OPL of MOG/CFA-injected EAE mice and CFA-injected control mice during the repolarization phase (120–180 s) in the presence ([+baf]) or absence ([−baf]) of bafilomycin-A1. In (**C**), the curves from (**B**) in the presence of bafilomycin A1 were subtracted from the curves in the absence of bafilomycin A1 ([−baf]-[+baf]) to obtain the values for endocytosis for CFA-injected control mice and MOG/CFA-injected EAE mice. Data points in (**C**) could be fitted best by a double-exponential curve from which the amplitudes of fast and slow endocytosis during the recovery phase were extracted (**D**). Values in (**D1**,**D2**) are means ± S.E.M. from amplitude 1 and amplitude 2 respectively. Unpaired Student’s *t*-tests were used to determine the statistical significance. N, number of mice; n, number of retinal slices used for optical recording in the presence/absence of bafilomycin A1; **, *p* ≤ 0.01; *, *p* ≤ 0.05.

Next, we analyzed whether compensatory post-stimulus endocytosis was also altered in photoreceptor synapses of MOG/CFA-injected EAE mice. In order to do that, we measured post-depolarization SypHy responses in the absence/presence of bafilomycin-A1 and determined endocytosis as the difference of fluorescence signals from the mean traces in the absence/presence of bafilomycin (Figure 8A). Bafilomycin-A1 was added to the depolarization solution (in the 60–120 s incubation window, see Figure 8A), and to the subsequent resting solution (120–160 s time window, see Figure 8A). In the latter time window, we measured endocytosis. Endocytosis in the post-stimulus repolarization phase ([−baf]-[+baf]) could be best fitted by a double-exponential curve (Figure 8C). From the double-exponential curves the fast and slow amplitudes (amplitude 1 and amplitude 2, resp.) were extracted (Figure 8(D1,D2)). Endocytosis in the post-depolarization recovery phase was significantly reduced in photoreceptor synapses of the OPL from MOG/CFA-injected early EAE mice in comparison to CFA-injected control mice (Figure 8C). Both amplitude 1 and amplitude 2 (amplitudes of fast and slow endocytosis) were significantly reduced in early EAE mice in comparison to littermate control-injected mice (Figure 8(D1,D2)). In the depolarization phase (60–120 s time window in Figure 8A) the SypHy curves recorded in the presence/absence of bafilomycin A1 nearly completely overlapped indicating that there is no significant endocytosis taking place in the depolarization phase at the selected conditions.

### 2.6. Decreased Enrichment of Dynamin3 at Photoreceptor Synapses in Early EAE Mice in Comparison to Control Mice

**Figure 9:**
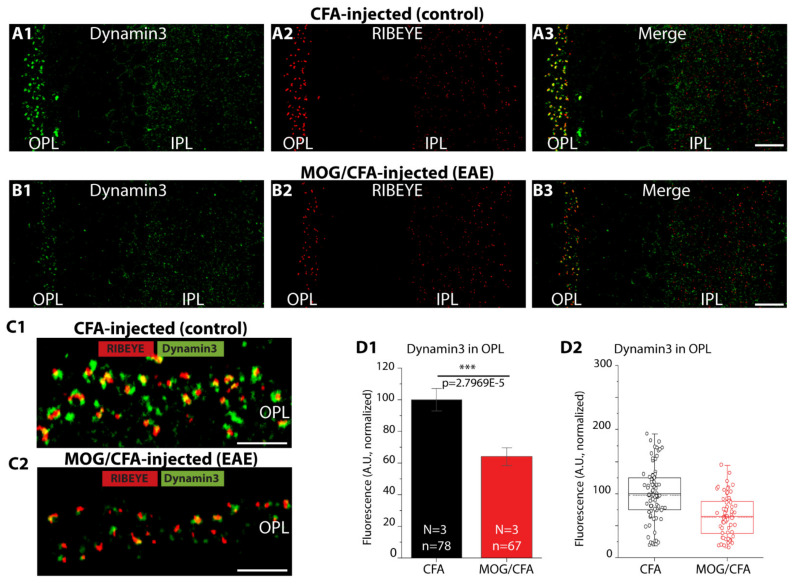

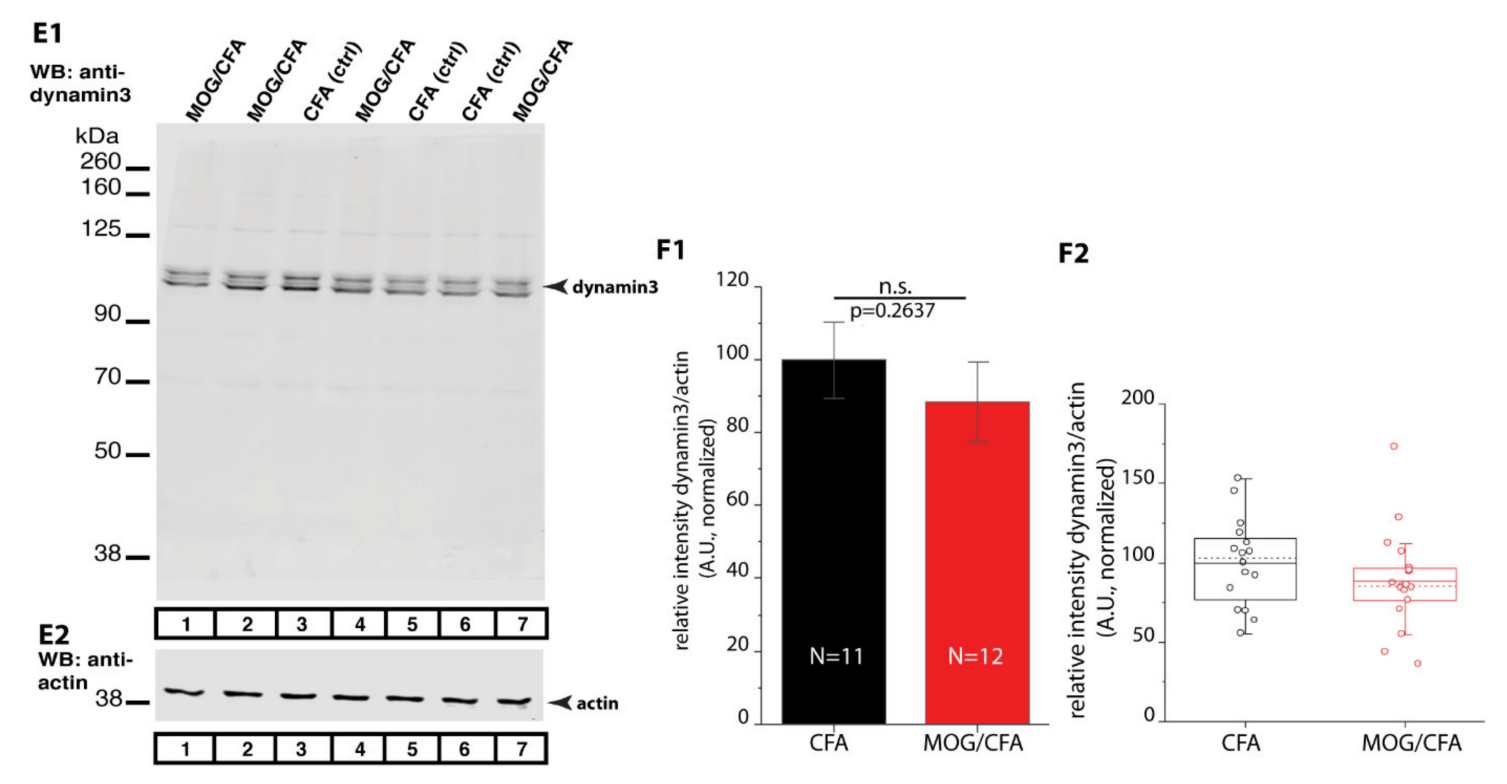
Decreased enrichment of dynamin 3 to photoreceptor synapses in the OPL of MOG/CFA-injected early EAE mice. (**A1**–**C2**) Semi-thin retina sections from CFA- and MOG/CFA-injected mice obtained on day 9 after injection were double-immunolabelled with anti-dynamin 3 and anti-RIBEYE antibody, as indicated. (**C1**,**C2**) Magnified views of the OPL from CFA-injected control and MOG/CFA-injected EAE mice that were double-immunolabelled with dynamin 3 and RIBEYE antibodies. (**D1**,**D2**) Quantification of the fluorescence intensity (integrated density) of dynamin 3 immunosignals in the OPL of MOG/CFA-injected and CFA-injected control mice. Values in (**D1**) are mean ± S.E.M. In the box-and-whiskers plots (**D2**) of the data from (**D1**), mean values are indicated by solid horizontal lines; median values by dotted horizontal lines. Boxes represent the 25th–75th percentiles of data points and whiskers are equal to 1.5 times of the interquartile range (IQR). Mann–Whitney U-test was applied to determine the statistical significance for fluorescence intensity (**D1**,**D2**). Abbreviations: OPL, outer plexiform layer; IPL, inner plexiform layer; S.E.M., standard error of the mean; N = number of mice; n = number of confocal images analyzed to quantify fluorescence intensity. Scale bars: 5 µm. (**E1**–**F2**) Western blot analyses of retinal lysates from MOG/CFA-injected EAE mice and CFA-injected control mice probed with anti-dynamin 3 antibody (**E1**). Actin served as loading control (**E2**). (**F1**,**F2**) Quantification of Western blot signals of dynamin 3 in MOG/CFA-injected and CFA-injected control. The total fluorescent intensity values of the WB bands were computed using Image Studio Lite software and normalized to the loading control (actin). The CFA-control was set to 100% to better evaluate the relative change in MOG/CFA-injected EAE vs. CFA-injected control mice. Values in (**F1**) are means ± S.E.M. In the box-and-whiskers plots (**F2**) of the data from (**F1**), mean values are indicated by solid horizontal lines; median values by dotted horizontal lines. Boxes represent the 25th–75th percentiles of data points and whiskers are equal to 1.5 times of the interquartile range (IQR). Unpaired Student’s *t*-test was used to determine the statistical significance. N, number of mice; ***, *p* ≤ 0.001; n.s., not significant.

We searched for possible underlying mechanisms for the decreased post-stimulus-endocytosis in EAE photoreceptor synapses. Dynamins are key proteins required for most forms of endocytosis at synapses [43,44,45,46,47,48,49]. Different types of endocytosis might occur at the synapse [50,51]. Since dynamin3 was found to be highly enriched in photoreceptor synapses of un-treated wildtype retina [52], we analyzed the distribution of dynamin3 in the OPL of MOG/CFA-injected EAE mice in comparison to CFA-injected control mice (Figure 9). We found a strong reduction of dynamin3 expression in the OPL of MOG/CFA-injected EAE mice in comparison to CFA-injected control mice (Figure 9A–C; for quantification Figure 9D). The decreased dynamin3 immunosignals were not due to a global reduction of dynamin3 expression, as judged by WB analyses (Figure 9E; for quantification, Figure 9F), but due to a reduced recruitment of dynamin3 to the synapse (see Section 3).

### 2.7. Basal (Resting) Synaptic Vesicle Cycling Is Increased in Photoreceptor Synapses of MOG/CFA-Injected EAE Mice

**Figure 10:**
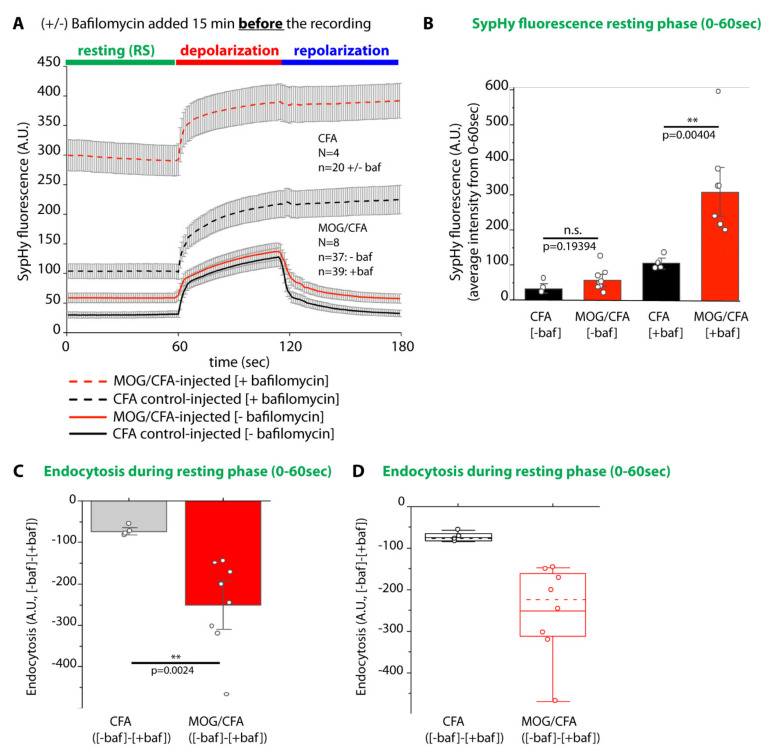
Basal endocytosis during the resting phase is higher in MOG/CFA-injected EAE mice compared to CFA-injected control mice. (**A**) Optical recording response of SypHy fluorescence in the OPL of MOG/CFA-injected EAE mice and CFA-injected control mice retinal slices on day 9 after injection. The slices were pre-incubated with or without bafilomycin A1 for 15 min before recording. (**A**) shows non-normalized response of SypHy fluorescence in the OPL of MOG/CFA-injected EAE mice and CFA-control injected mice in the presence and absence of bafilomycin A1. (**B**) Basal average level of SypHy fluorescence during resting stage (0–60 sec) in the presence and absence of bafilomycin A1 in MOG/CAF-injected EAE mice and CFA-injected control mice. (**C**) Total endocytosis during the resting phase (0–60 s) was determined by subtracting the basal fluorescence in the presence of bafilomycin A1 [+baf] from the basal fluorescence in the absence of bafilomycin A1 [−baf]; i.e., ([−baf]-[+baf]). (**D**) In the box-and-whisker plot from the data in (**C**), mean values are represented by solid horizontal line; median by dotted horizontal line. Boxes represent the 25th–75th percentile of the data points and whiskers are equal to 1.5 times of the IQR. Mann–Whitney U test was applied to obtain statistical significance in (**B**) and unpaired Student’s *t*-test in (**C**). N = number of mice; n = number of retinal slices used for the optical recording in the presence or absence of bafilomycin A1; n.s., not significant; **, *p* ≤ 0.01; RS, resting solution.

A recent study demonstrated an increase of basal (resting) Ca^2+^ concentration in the presynaptic rod photoreceptor terminals of MOG/CFA-injected EAE mice [25]. Therefore, we tested whether excocytic and endocytic vesicle cycling, that critically depends upon Ca^2+^, is altered already under resting conditions in photoreceptor synapses of MOG/CFA-injected EAE mice in comparison to CFA-injected control mice (Figure 10). For this purpose, we measured the basal vesicle cycling in the absence/presence of bafilomycin A1 (0–60 s time window in Figure 10A).

We noted that basal SypHy fluorescence signals (0–60 s time window in Figure 10A) in photoreceptor synapses of MOG/CFA-injected EAE mice were much higher than in CFA-injected control mice in the presence of bafilomycin (Figure 10A,B), indicating an increased level of vesicle exocytosis in EAE photoreceptor synapses under resting conditions. This increased basal vesicle exocytosis is likely a consequence of the previously reported increased levels of resting presynaptic Ca^2+^ ([25]; see Section 3). Basal exocytosis in photoreceptor synapses was about three-fold higher in MOG/CFA-injected EAE mice in comparison to CFA-injected control mice, as judged by SypHy recording in the presence of bafilomycin A1 (Figure 10A).

We also calculated the amplitudes of basal endocytosis from the fluorescence values obtained in the absence and presence of bafilomycin A1. Endocytosis under resting conditions was significantly elevated in photoreceptor synapses of MOG/CFA-injected EAE mice in comparison to CFA-injected littermate control mice (Figure 10C,D).

### 2.8. Elevated De-Phosphorylation of Dynamin1 in Photoreceptor Synapses of MOG/CFA-Injected EAE Mice

**Figure 11:**
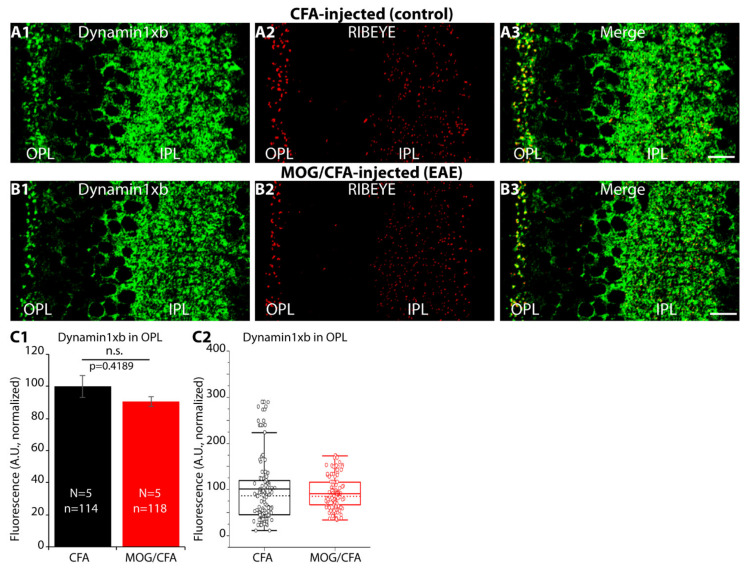

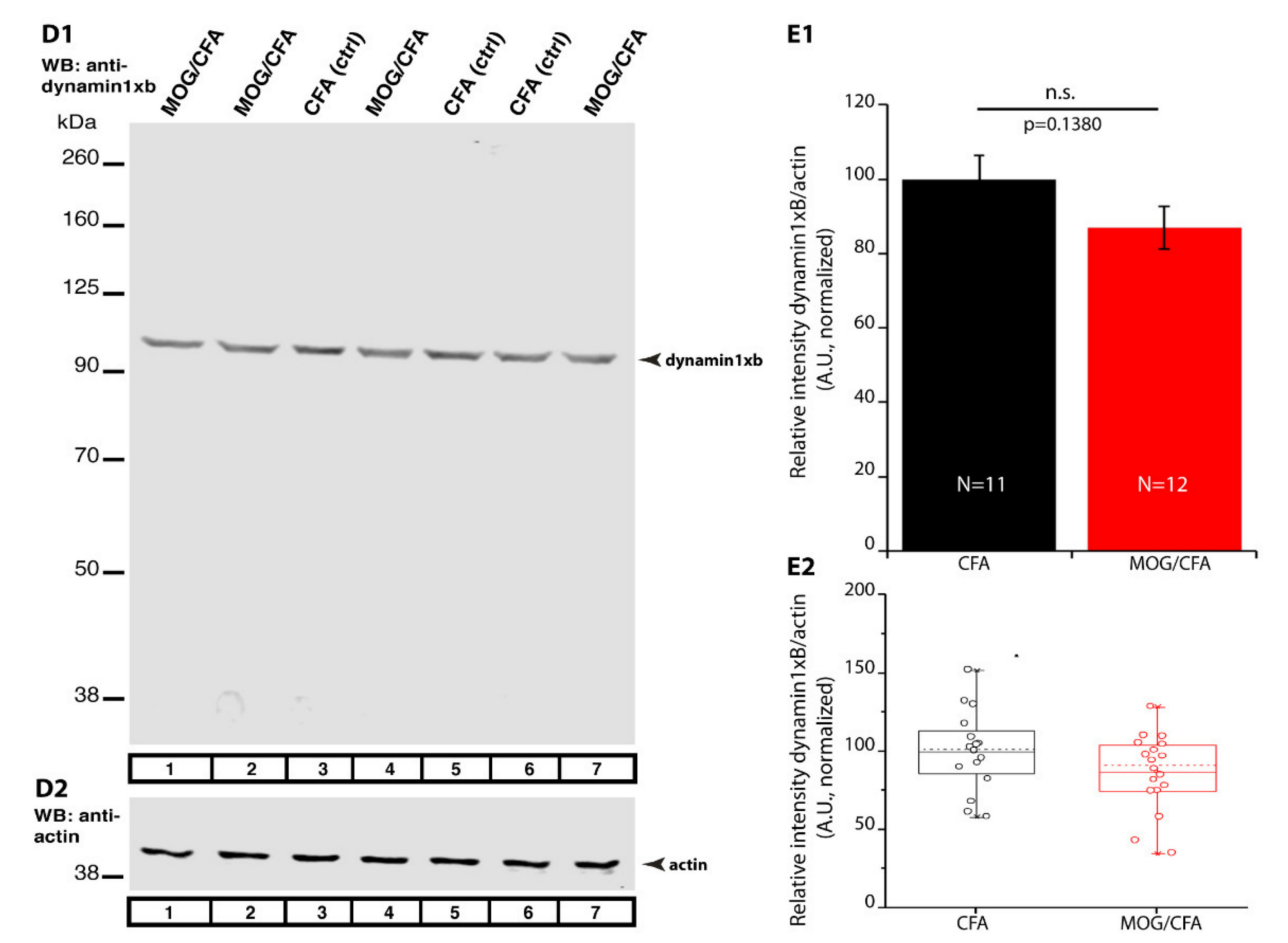
Dynamin1xb expression in the OPL does not differ between MOG/CFA-injected EAE mice and CFA-injected control mice. (**A1**–**B3**) Retina sections (0.5 µm thickness) from CFA- and MOG/CFA-injected mice retina obtained on day 9 after injection were double-immunolabelled with dynamin1xb and RIBEYE (U2656) antibody. (**C1**,**C2**) Quantification of the fluorescence intensity (integrated density) of dynamin1xb immunosignals in the OPL of MOG/CFA-injected EAE mice and CFA-injected control mice. Values in (**C1**) are mean ± S.E.M. In the box-and-whiskers plots (**C2**) of the data from (**C1**), mean values are indicated by solid horizontal lines; median values by dotted horizontal lines. Boxes represent the 25th–75th percentiles of data points and whiskers are equal to 1.5 times of the interquartile range (IQR). Mann–Whitney U-test was applied to determine the statistical significance for fluorescence intensity. Abbreviations: OPL, outer plexiform layer; IPL, inner plexiform layer; S.E.M., standard error of the mean; N = number of mice; n = number of confocal images analyzed to quantify fluorescence intensity. Scale bars: 5 µm. (**D1**–**E2**) Western blot analyses of dynamin1xb expression in retinal lysate obtained from MOG/CFA-injected EAE and CFA-injected (control) mice retina, isolated on day 9 after the injection. 30 µg of protein was loaded in each lane. Blots were probed with dynamin1xb antibody (**D1**) and for loading control an actin antibody was used (**D2**). (**E1**,**E2**) Quantification of the intensity of dynamin1xb WB bands. The band intensity of the dynamin1xb in each lane was normalized with actin control in the same lane. To evaluate the relative change in dynamin1xb between MOG/CFA vs. CFA (control), the CFA (control) was set to 100%. Values in (**E1**) are means ± S.E.M. In the box-and-whiskers plots (**E2**) of the data from (**E1**), mean values are indicated by solid horizontal lines; median values by dotted horizontal lines. Boxes represent the 25th–75th percentiles of data points and whiskers are equal to 1.5 times of the interquartile range (IQR). Unpaired Student’s *t*-test was used to determine the statistical significance in (**E1**,**E2**). N, number of mice; n.s., not significant.

Since we observed an elevated level of basal endocytosis in photoreceptor synapses of early EAE mice, we searched for possible underlying molecular mechanisms and focused on dynamin1xb [53,54]. Dynamin1xb is highly expressed in synapses of the central nervous system including the retina [55]. We confirmed that dynamin1xb is highly enriched in retinal synapses located in the OPL and IPL (Figure 11(A1–B3)), as previously published [55]. Quantitative analyses showed that the dynamin1xb immunofluorescence signals did not differ between MOG/CFA-injected EAE animals and CFA-injected control animals (Figure 11(C1,C2)). Similarly, also in Western blot analyses we did not observe a difference in the expression of dynamin1xb in retinal lysates isolated from MOG/CFA-injected mice in comparison to CFA-injected control mice (Figure 11(D1–E2)).

**Figure 12:**
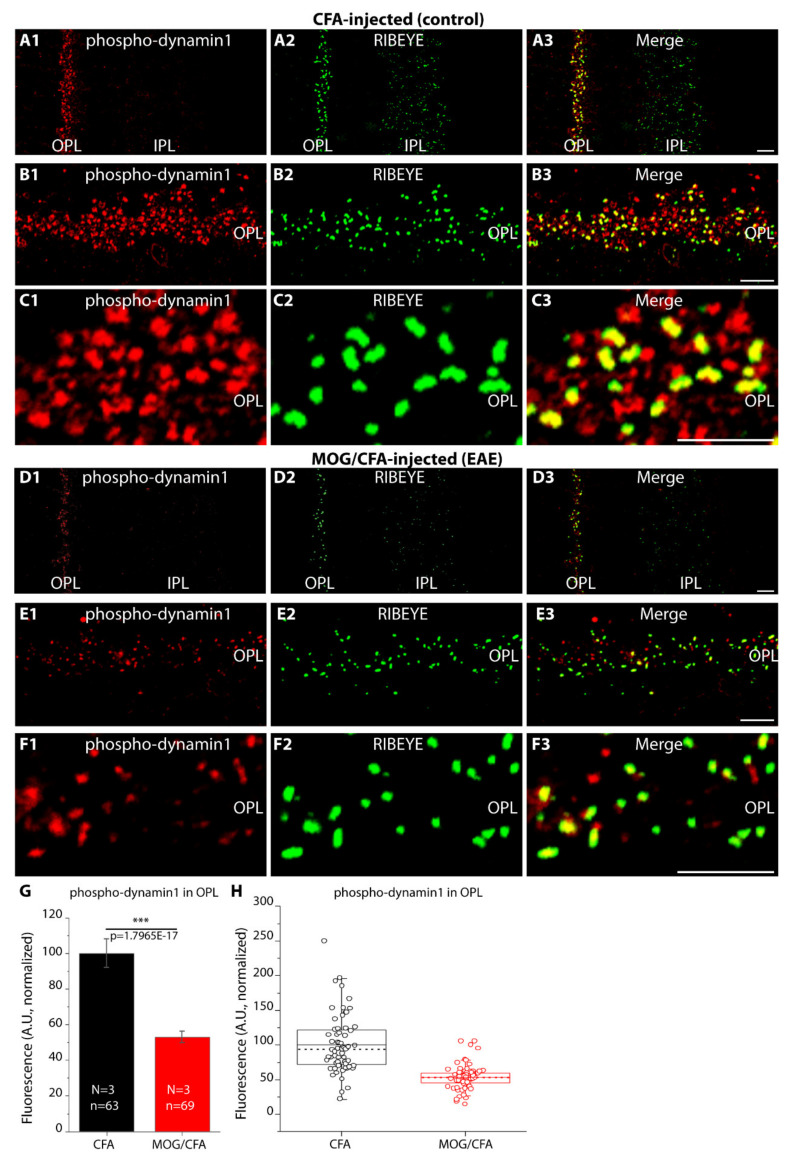
Phospho-dynamin1 immunosignals in photoreceptor synapses of MOG/CFA-injected early EAE mice are reduced in comparison to control mice. (**A1**–**F3**) Retina sections (0.5 µm thickness) from CFA- and MOG/CFA-injected mice obtained on day 9 after injection were double immunolabelled with phospho-dynamin1 and RIBEYE antibody (clone 2D9), as indicated in the figure. (**C1**–**C3**,**F1**–**F3**) magnified views of the OPL immuno-labelled with anti-phospho-dynamin1 and anti-RIBEYE. (**G**,**H**) Quantification of fluorescence intensity (integrated density) of phospho-dynamin1 immunosignals in the OPL of MOG/CFA-injected and CFA-injected control mice. Values in (**G**) are means ± S.E.M. In the box-and-whiskers plots (**H**) of the data from (**G**), mean values are indicated by solid horizontal lines; median values by dotted horizontal lines. Boxes represent the 25th–75th percentiles of data points and whiskers are equal to 1.5 times of the interquartile range (IQR). Mann–Whitney U-test was applied to determine the statistical significance for fluorescence intensity in (**G**,**H**). (**C1**–**C3**,**F1**–**F3**) High magnification confocal images of the immunolabelled OPL indicate enrichment of phospho-dynamin1 at the ribbons but also expression of phospho-dynamin1 in some distance from the ribbons. Abbreviations: OPL, outer plexiform layer; IPL, inner plexiform layer; S.E.M., standard error of the mean; N, number of mice; n, number of confocal images analyzed to quantify fluorescence intensity; ***, *p* ≤ 0.001. Scale bars: 5 µm (**A3**–**F3**).

**Figure 13:**
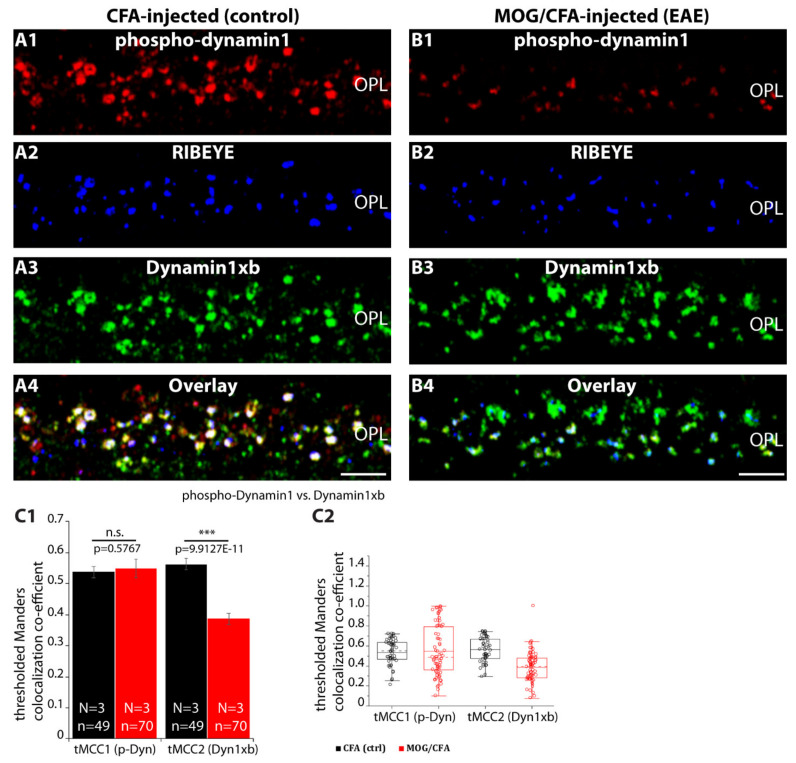
Triple immunolabelling of retinal semithin section from CFA-injected control and MOG/CFA-injected EAE mice. (**A1**–**B4**) 0.5 µm semi-thin sections from CFA-injected (control) mice and MOG/CFA-injected EAE mice were triple-immunolabelled with sheep polyclonal anti-p-dynamin1, rabbit polyclonal anti-RIBEYE (U2656), and mouse monoclonal anti-dynamin1xb. (**C**) Manders colocalization coefficients show the degree of colocalization between p-dynamin1 and dynamin1xb in the photoreceptor ribbon synapse in the OPL of CFA-injected control mice and MOG/CFA injected EAE mice. Values in (**C1**) are means ± S.E.M. In the box-and-whisker plots (**C2**) of the data from (**C1**), mean value are represented by solid horizontal lines; median by dotted horizontal lines. Boxes represent the 25th–75th percentile of the data points and whiskers are equal to 1.5 times of the IQR. Mann–Whitney U-test was used to determine the statistical significance because data were non-normally distributed in (**C1**,**C2**). Abbreviations: OPL, outer plexiform layer; N, number of mice; n, number of confocal images used for colocalization analysis; ***, *p* ≤ 0.001; tMCC, thresholded Manders colocalization co-efficient. Scale bars: 5 µM (**A4**,**B4**).

Dynamin1 (and dynamin3) are regulated by phosphorylation [45,56,57,58,59,60]. Therefore, we analyzed the expression pattern of phospho-dynamin1 using a well-characterized anti-phospho-dynamin1 antibody. We found phospho-dynamin1 highly enriched in photoreceptor synapses of CFA-injected control mice analyzed 9 days after injection (Figure 12(A1–C3)). The immunosignal for phospho-dynamin1 in the OPL of MOG/CFA-injected EAE littermate mice was strongly reduced in comparison to littermate CFA-injected control mice (Figure 12(D1–F3); for quantification, Figure 12G,H). Phospho-dynamin1 signals were found in close proximity to the photoreceptor ribbons but also at non-ribbon sites (Figure 12C,F), as seen by the distribution of the respective immunosignals (Figure 13C). Increased levels of de-phosphorylation of dynamin1 in photoreceptor synapses of MOG/CFA-injected EAE mice were also confirmed by triple immunolabelling experiments of anti-phospho-dynamin1 with anti-dynamin1xb and anti-RIBEYE (Figure 13). Phospho-dynamin1 was less enriched at the photoreceptor synapses whereas the levels of dynamin1xb remained unchanged (Figure 13(A1–B4)). As a consequence of the lower levels of phospho-dynamin1 in MOG/CFA-injected EAE mice, dynamin1xb showed a decreased Manders co-localization coefficient (tMCC2) with phospho-dynamin1 in EAE (Figure 13(C1,C2)).

**Figure 14:**
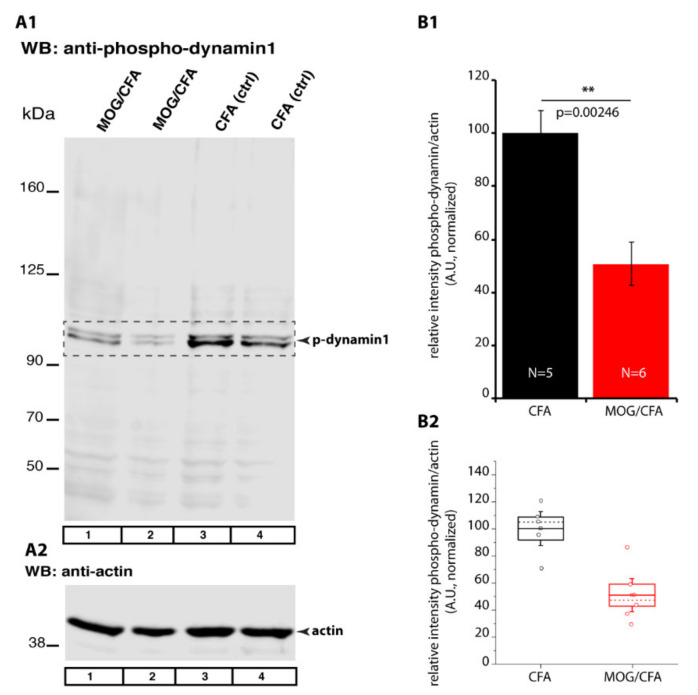
Phospho-dynamin1 expression in the retina of MOG/CFA-injected EAE mice is reduced (WB analyses). (**A1**,**A2**) Retinal lysates isolated from MOG/CFA-injected EAE mice and CFA-injected control mice were probed by WB analyses with anti-phospho-dynamin1 antibody (**A1**) and anti-actin antibody (loading control) (**A2**). (**B1**,**B2**) Quantification of phospho-dynamin1 WB bands in MOG/CFA-injected and CFA-injected control using the Li-Cor system. The total fluorescent intensity values of the bands were computed using Image Studio Lite software and normalized to the loading control (actin). The CFA-control was set to 100% to better evaluate the relative change in MOG/CFA-injected EAE vs. CFA control mice. Values in (**B1**) are means ± S.E.M. In the box-and-whiskers plots (**B2**) of the data from (**B1**), mean values are indicated by solid horizontal lines; median values by dotted horizontal lines. Boxes represent 25th–75th percentiles of data points; whiskers are equal to 1.5 times of the interquartile range (IQR). Unpaired Student’s *t*-test was used to determine statistical significance. N, number of mice; **, *p* ≤ 0.01; p-dynamin1, phospho-dynamin1.

**Figure 15:**
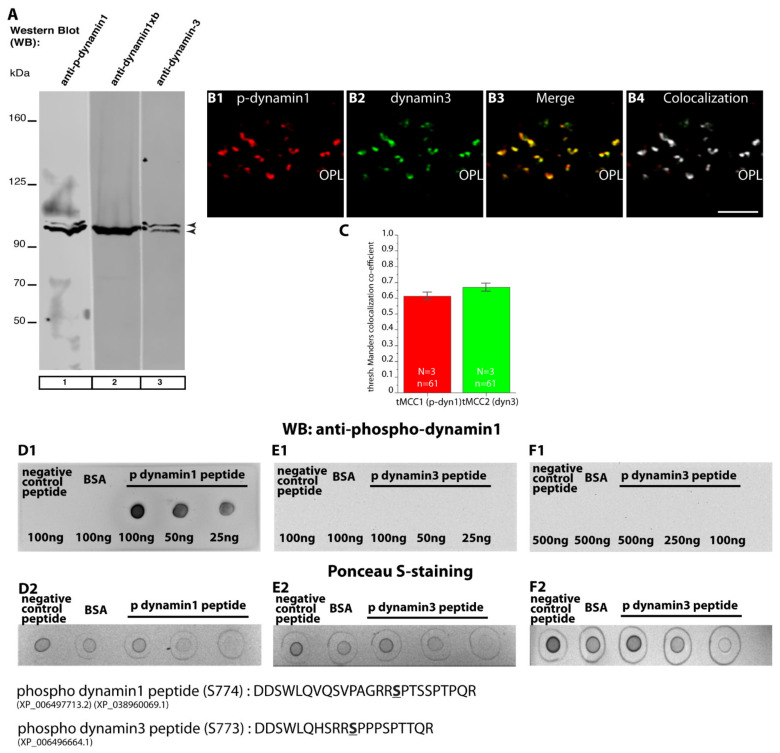
Characterization of different dynamin antibodies and their possible cross-reactivity and co-localization. (**A**) Western blot (WB) analyses of the indicated dynamin antibodies on retinal lysates of un-treated wild-type C57BL/6 mice. (**B1**–**B4**) Confocal images of semi-thin sections from untreated wild-type retina showing the OPL that was double-immunolabelled with anti-phospho-dynamin1 and anti-dynamin 3, as indicated. (**C**) Thresholded Manders colocalization coefficients show the degree of colocalization between phospho-dynamin1 and dynamin3 in the OPL. (**D1**–**F2**) Dot blot analyses were performed to characterize the anti-phospho-dynamin1 antibody for its reactivity with the indicated peptides (p-dynamin1 and p-dynamin3) cross-linked to BSA. p-dynamin1 peptide (DDSWLQVQSVPAGRR**S**PTSSPTPQR; phospho-serine is underlined and indicated in bold) (**D1**,**D2**) and p-dynamin3 peptide (DDSWLQHSRR**S**PPPSPTTQR; phospho-serine is underlined and indicated in bold) (**E1**–**F2**) were spotted on nitrocellulose membrane. An unrelated control peptide (CENEIQDLLRAKRELESKLQRLQAQG) cross-linked to BSA as well as BSA only (with no peptide cross-linked to it) were used as negative controls for non-specific antibody binding (**D1**–**F2**). (**D1**) The anti-p-dynamin 1 antibody strongly reacted with the p-dynamin1 peptide (DDSWLQVQSVPAGRR**S**PTSSPTPQR). (**E1**,**F1**) The anti-p-dynamin1 antibody did not cross-react with the p-dynamin3 peptide (**E1**), the negative control peptide (**F1**), or BSA alone (**D1**,**E1**,**F1**). (**D2**,**E2**,**F2**) Ponceau-S staining of the respective dot blot nitrocellulose membranes with the spotted cross-linked peptides (spotting controls). Abbreviations: OPL, outer plexiform layer; tMCC, thresholded Manders colocalization co-efficient. Scale bar: 5 µm (**B**).

We also analyzed phospho-dynamin1 levels by Western blot (WB) experiments (Figure 14(A1–B2)). In WB analyses of retinal lysates isolated from MOG/CFA-injected mice, we found a significant reduction of phospho-dynamin1 expression in comparison to retinal lysates isolated from CFA-injected mice (Figure 14(A1); for quantification, see Figure 14(B1,B2)). Actin served as loading controls in the WB experiments to ensure equal protein loading (Figure 14(A2)). In WB analyses, the anti-phospho-dynamin1 antibody showed a similar band pattern as anti-dynamin3 (Figure 15A). Furthermore, dynamin3 is similarly enriched in photoreceptor synapses as dynamin1, as judged by IF (Figure 15(B1–B4),C), raising the possibility that the anti-phospho-dynamin1 antibody might also cross-react with dynamin3. But dot blot analyses with the respective peptides (Figure 15(D1–F2)) demonstrated that the anti-phospho-dynamin1 antibody is specific for dynamin1. The anti-phospho-dynamin1 antibody only reacted with the dynamin1 phospho-peptide (Figure 15(D1)) but not with the corresponding homologous dynamin3 phospho-peptide (Figure 15(E1,F1)) nor with negative control peptides (Figure 15(D1–F1)). From this, we conclude that the applied phospho-dynamin1 antibody is specific for phospho-dynamin1 (phosphorylated at Ser774).

## 3. Discussion

In previous studies [24,25], we observed dysfunctions of retinal synapses in the EAE mouse model of multiple sclerosis before obvious signs of optic nerve demyelination/optic neuritis [24]. [24] showed that RIBEYE immunosignals were decreased in rod photoreceptor synapses of EAE mice at an early pre-clinical stage. In the present study, we found that the number of ribbons were unchanged in the total population of rod photoreceptor synapses in these early EAE mice. Instead, we observed that synaptic ribbons in rod photoreceptor synapses are smaller in size in early EAE mice in comparison to littermate control mice, as judged by the quantitative 3D SR-SIM analyses and by transmission electron microscopy. Rod synaptic ribbon were shorter in height and shorter in length. The ribbon length denotes the portion of the ribbon that runs in parallel to the active zone (Figure 1(A2)). This part is physically anchored to the active zone [61]. The decrease in rod ribbon length in early EAE observed in the present study correlates well to the previously reported decrease in the length of active zone protein clusters in early EAE [25]. These active zone protein clusters contain RIM2 and voltage-gated Ca^2+^-channels [25]. Various previous studies showed a strong inter-dependence between synaptic ribbons and presynaptic Ca^2+^-channels [62,63,64,65,66,67,68,69,70]. Therefore, the previously described decrease in the size of the active zone protein/Cav-channel clusters at rod photoreceptor synapses in early EAE [25] and the shorter synaptic ribbons likely depend on each other. Altered synaptic ribbons were also found to be associated with changes in presynaptic Ca^2+^ in other systems [42,71,72,73].

What could be the mechanism that results in smaller rod photoreceptor synaptic ribbons in early EAE? As outlined in the introduction, RIBEYE is a main building block of synaptic ribbons [29,30] consisting of a N-terminal A-domain and a carboxyterminal B-domain. The carboxyterminal B-domain binds NAD(H) and the binding of NADH/NAD^+^ affects RIBEYE-RIBEYE interactions [74]. Recently, it has been shown that the NADH/NAD^+^ redox ratio can modulate the ribbon size [75,76]. The NADH/NAD^+^ ratio is crucially influenced by mitochondria and by cytosolic Ca^2+^ levels [77,78,79,80,81,82,83,84,85]. Recently, it was shown that basal presynaptic Ca^2+^ levels at mouse photoreceptor synapses are altered (increased) in early EAE [25]. Therefore, it appears possible that dysfunctional presynaptic Ca^2+^ in early EAE can influence synaptic ribbon size via changes in the NADH/NAD^+^ redox ratio. Of note, mitochondrial dysfunctions have been documented in multiple sclerosis [86,87,88]. Auto-antibodies directed against CASPR1, a synaptic adhesion protein located close to the active zone/synaptic ribbon, could also play a role [24]. Auto-antibodies against CASPR1 appear early on in pre-clinical EAE [24] and could affect active zone size and subsequently the synaptic ribbon. Clearly, further analyses are required to discriminate between these possibilities.

Interestingly, loss or disassembly of synaptic ribbons was also observed in other types of ribbon synapse dysfunctions, e.g., in the inner ear (e.g., [41,76,89,90,91]) emphasizing the pathophysiological relevance of alterations in synaptic ribbons. Likewise, differences in ribbon size have been recognized as important determinants of the properties of individual ribbon synapses in inner ear hair cells [28,92,93,94]. Differences in ribbon size have been previously also described in retinal synapses reflecting different states of functional activity [28,68,95,96,97,98].

The decrease in ribbon size in rod photoreceptor synapses of early EAE mice was accompanied by complex alterations of synaptic vesicle cycling. We observed a significant decrease in depolarization-evoked vesicle exocytosis and also a decrease in compensatory post-stimulus endocytosis (Figure 7 and Figure 8). Decreased depolarization-evoked vesicle exocytosis has already been previously observed on day 9 after injection [24] and is likely based on the decreased ribbon-associated vesicle pools that were observed in the present study. The size of these vesicle pools are important determinants of synaptic transmission at ribbon synapses. We confirm these previous findings and demonstrate that these disturbances of synaptic vesicle exocytosis are already present as early as on day 7 after injection. We also checked earlier time points because a recent study showed that disturbances of presynaptic Ca^2+^ homeostasis are already present on day 7 after injection [25]. Remarkably, the effect of MOG/CFA injection on synaptic vesicle exocytosis in early EAE, as judged by optical SypHy recording was already maximally strong on day 7 after injection and did not further increase on day 8 and day 9 after injection (Figure 7D). This finding suggests that future analyses have to consider even earlier time points, i.e., earlier than day 7 after injection. This very early onset of changes in exocytosis in MOG/CFA-injected mice is very remarkable because all analyzed time points are within the pre-clinical phase. The early synaptic changes might be related to the breakdown of the blood-brain-barrier (BBB), the infiltration of immune cells (e.g., macrophages, activated T cells, auto-reactive B cells) into the CNS and activation of glia cells. Inflammatory infiltrates occur early on in MS/EAE [2,3,4]. Infiltrating immune cells and activated glia cells secrete various cytokines that could well affect synapse function [2,3,4].

The observed decrease in depolarization-evoked exocytosis in early EAE is likely caused by alterations of the active zone. Active zone alterations in early EAE were recently identified in photoreceptor synapses [25]. The active zones of photoreceptor synapses in early EAE showed a decreased enrichment of important active zone proteins, including voltage-gated Cav-channels that trigger depolarization-evoked exocytosis [25]. The length of synaptic ribbons and active zones are tightly correlated [68]. Both structures are physically linked to each other along the membrane-anchored end of the ribbon [61]. Therefore, the smaller active zone protein clusters in early EAE [25] and the decreased ribbon length, identified in the present study likely depend on each other and are part of a common mechanism. At this early stage, no obvious signs of optic nerve demyelination were observed [24] arguing that the synapse alterations are not secondary events caused by optic nerve demyelination. Our data suggest that the synaptic ribbon plays a role for exocytic and endocytic vesicle trafficking in early EAE photoreceptor synapses.

Remarkably, we also found a decrease in post-stimulus endocytosis, i.e., endocytosis that occurred after depolarization-evoked exocytosis, in EAE photoreceptor synapses (Figure 8(C–D2)). The reason for this decreased post-stimulus endocytosis remains to be elucidated. Decreased post-depolarization endocytosis in early EAE photoreceptors correlated with a decreased synaptic enrichment of dynamin3 indicating that the decreased recruitment of dynamin3 to photoreceptor terminals in early EAE could account for the decreased post-stimulus endocytosis. Possibly, ribbon size/active zone length also affects the peri-active zone/peri-active zone properties. The peri-active zone surrounds the active zone and has been proposed to be the preferential site of vesicle endocytosis and of exocytosis/endocytosis coupling [99,100,101,102,103,104], as also suggested for photoreceptor synapses [105].

In contrast to the decreased evoked responses (i.e., depolarization-evoked exocytosis and post-stimulus endocytosis), basal vesicle turnover appeared to be operating at elevated levels in early EAE photoreceptor synapses. This was shown by SypHy imaging in the absence/presence of bafilomycin-A1 (Figure 10). The reason for this increased basal synaptic vesicle turnover most likely is the previously reported increased levels of basal, presynaptic Ca^2+^ [25]. Vesicle fusion in rod photoreceptor synapses is exquisitely sensitive to Ca^2+^ [106,107]. Elevated basal presynaptic Ca^2+^ likely can also account for the increased basal endocytosis, as previously observed also in other synapses [104,108,109,110].

Dynamin1xb is an activity-dependent splice variant of dynamin1 with a docking site for the Ca^2+^-dependent phosphatase calcineurin [53,54]. Elevated Ca^2+^ will activate the Ca^2+^-dependent phosphatase calcineurin leading to dynamin1 de-phosphorylation (at Ser774) and activation [54,56,111,112,113,114,115,116,117,118,119,120]. De-phosphorylation of dynamin1 triggers activity-dependent endocytosis [113,114,115,116,117,118]. Dynamin1xb and calcineurin, as well as other components of activity-dependent endocytosis (syndapin) are highly enriched in the presynaptic terminals of rod photoreceptors close to the synaptic ribbon [105].

In the present study, we found in both WB and IF experiments that dynamin1xb levels did not differ between photoreceptor synapses of MOG/CFA-injected EAE mice in comparison to CFA-injected littermate control mice (Figure 11). But dynamin1xb was more strongly de-phosphorylated, and thus more strongly activated, in MOG/CFA-injected retinas than in CFA-injected littermate retinas as demonstrated by WB analyses (Figure 14). IF data confirmed lower levels of phospho-dynamin1 in photoreceptor synapses of MOG/CFA-injected EAE retinas in comparison to the respective controls (Figure 12 and Figure 13). Interestingly, proteome analyses identified down-regulation of proteins involved in endocytosis in the mouse EAE model [121,122].

In conclusion, our study revealed smaller synaptic ribbons and ribbon-associated vesicle pools in photoreceptor synapses and complex alterations of synaptic vesicle cycling at an early stage in the EAE mouse model of multiple sclerosis. Ca^2+^ dysfunctions most likely play a central role. Calcium homeostasis is also known to be altered in multiple sclerosis patients [11,123,124]. Future analyses will be required to further investigate the relevance of early synaptic changes observed in EAE for human patients that suffer from multiple sclerosis.

## 4. Materials and Methods

### 4.1. Animals

Experiments were performed with 10–12 weeks old female C57BL/6J mice (20–25 g body weight) that were injected either with CFA or MOG/CFA (see below). All analyses were done in the pre-clinical phase of EAE (from day 7 to day 9 after injection), as indicated in the specific experiments. Mice were kept at standard light/dark cycles and were provided with water and standard food ad libitum. All animal procedures were reviewed and approved by the local animal authorities and performed according to the German Animal Protection Law. Mice were killed in a time window between noon and 4 pm. Mice were handed to the experimenter without revealing whether the respective mouse was MOG/CFA-injected or CFA-injected. SypHy mice were maintained as previously described [24].

### 4.2. Solutions

Resting solution (RS): 132 mM NaCl, 3 mM KCl, 1 mM MgCl_2_x6H_2_O, 2 mM CaCl_2_, 10 mM HEPES, pH 7.4, 10 mM sodium pyruvate, 10 mM glucose (osmolality 305–315 mOsmol/kg).

Low Ca^2+^ solution (LCS): 132 mM NaCl, 3 mM KCl, 1 mM MgCl_2_x6H_2_O, 0.5 mM CaCl_2_, 10 mM HEPES, pH 7.4, 10 mM sodium pyruvate, 10 mM glucose (osmolality 305–315 mOsmol/kg).

Depolarization solution (DS): 85 mM NaCl, 50 mM KCl, 1 mM MgCl_2_x6H_2_O, 2 mM CaCl_2_, 10 mM HEPES, pH 7.4, 10 mM sodium pyruvate, 10 mM glucose (osmolality 305–315 mOsmol/kg).

### 4.3. Bafilomycin

Bafilomycin A1 (Santa Cruz; sc-201550A) was stored frozen in 2.5 mM aliquots (in DMSO) and diluted before use to 5 μM (final concentration) in DS or RS for optical recording, as indicated. For vehicle control experiments, 0.1% DMSO in DS or RS, as indicated, but without bafilomycin was used.

### 4.4. Induction of Experimental Autoimmune Encephalomyelitis (EAE)

Experimental autoimmune encephalomyelitis (EAE) was induced exactly as previously described [24,25]. Female 10–12 weeks old C57BL/6J mice (20–25g body weight) were injected subcutaneously into the axilla and groin with encephalitogenic MOG_35–55_ peptide of mouse myelin oligodendrocyte glycoprotein (MEVGWYRSPFSRVVHLYRNGK) contained in a ready-to-go suspension from Hooke laboratories (MOG_35–55_/CFA Emulsion PTX, Hooke Laboratories, Lawrence, MA, USA; #EK-2110) or with self-made suspensions (prepared exactly as previously described [24]. To increase blood-brain barrier permeability, 200 ng of pertussis toxin (PTX) from *B. pertussis* (List Biological Laboratories/Biozol (Eching, Germany); #181) was injected intraperitoneally in a volume of 100 μL sterile glycerol buffer on the same day (day 0, 1–2 h after MOG_35–55_ peptide injection) and also on the subsequent day (day 1, 16–20 h after first PTX injection). Control animals were injected with CFA only, i.e., without MOG_35–55_ peptide (Hooke Laboratories, Lawrence. MA, USA; CFA control kit # CK-2110). All other treatments for the control injections, e.g., pertussis toxin injections, were done identically as described for MOG/CFA injection. Subsequent analyses of the injected animals were done blindly, i.e., with the experimenter being not aware whether a mouse was MOG/CFA-injected or CFA (control)-injected. All analyses were done in the pre-clinical phase of EAE, as indicated in the specific experiments.

### 4.5. Immunolabelling of Resin Sections of the Retina

Retinas were dissected within 5 min post-mortem and processed for Epon embedding as previously described [24,25,37,68,105,125]. In brief, tissue samples were first flash-frozen in liquid nitrogen-cooled isopentane. Subsequent lyophilization of the frozen tissue was performed at a vacuum of ≈10^−7^ mbar. During this process the tissue was continuously cooled with liquid nitrogen. The vacuum was generated with a TCP270 turbomolecular pump (Arthur-Pfeiffer-Vacuumtechnik, Wetzlar/Aßlar, Germany) controlled by a PKG020 Pirani-gold cathode gauge control unit and an oil diffusion pump as pre-pumping unit (type DUO 004B; Arthur-Pfeiffer-Vacuumtechnik, Wetzlar/Aßlar, Germany). Samples were lyophilized in liquid nitrogen for ≈24 h. Afterwards, samples were equilibrated to room temperature, infiltrated with Epon resin, and degassed for ≈24 h to ensure complete penetration with Epon. Curing of the resin-embedded samples was done at 60 °C for ≈24 h. Immunolabelling was performed on 0.5-μm-thin resin sections for confocal microscopy, as previously described [24,25,37,68,105,125]. Prior to immunolabelling resin was removed with sodium methanolate, as described [24,25,37,68,105]. After removal of the resin, sections were incubated with specified primary antibodies at the indicated dilutions (4 °C overnight). For double-immunolabelling analyses, sections were incubated simultaneously with the two primary antibodies (that were generated in two different species). After several washes with PBS to remove unbound primary antibodies, bound primary antibodies were detected by incubation with corresponding secondary antibodies conjugated to the indicated fluorophores (1:1000 dilution; 2 h at room temperature). For triple-immunolabelling analyses, sections were incubated simultaneously with the three primary antibodies (generated in three different animal species: sheep, mouse, rabbit). After several washes with PBS to remove unbound primary antibodies, bound primary antibodies were detected by incubation with corresponding secondary antibodies conjugated to the indicated fluorophores (1:1000 dilution; 2 h at room temperature). Immunolabelled sections were embedded in N-propyl gallate antifade [24,25,37,68,105]. Negative controls were done by omitting primary antibodies while all other steps were performed the same. Further controls were performed for the double- and triple immunolabelling experiments by setting individual laser power lines to zero with unchanged detection settings. These controls were done to make sure that the immunosignal in the channel of interest does not result from a bleed-through from the neighboring detection channels (“bleed through controls”). The list of primary and secondary antibodies is given in Table 1 and Table 2.

### 4.6. Confocal Microscopy and Quantitative Analyses of Immunosignals

Confocal microscopy was performed as previously described [24,25,37,68,105,125]. For confocal microscopy, an A1R confocal microscope system (Nikon; Düsseldorf, Germany) equipped with 488 nm and 561 nm laser excitation lines and the NIS Elements software (NIS Elements AR 3.2, 64 bit; Nikon; Düsseldorf, Germany) was used. Images were acquired with a 60×/1.40 N.A. oil objective. For quantitative analyses, confocal images of experimental and control retinas were acquired under identical conditions by using the “re-use” settings option of the NIS elements software every time to keep the same acquisition conditions for CFA-injected and MOG/CFA-injected samples, as previously described [24,25,37,68]. Quantitative analyses were performed in a blinded manner with the experimenter not knowing the identity of the samples. Colocalization analysis was done as described in [37]. Briefly, the colocalization threshold plugin of NIH ImageJ [127,128] which relies on the Costes automatic thresholding method [129] was used to calculate thresholded Mander’s co-localization coefficients [130,131]. The colocalization image in (Figure 15(B4)) was generated by applying the “Show Colocalization Pixels” tool in the colocalization threshold plugin of NIH ImageJ. Fluorescence intensity of the immunolabelled structures in the outer plexiform layer (OPL) was measured as integrated density with NIH ImageJ [127,128], as previously described [24,25,37,68]. The region of interest (ROI) was determined by the RIBEYE immunosignals that served as reference to define the location of photoreceptor synapses in the OPL [29]. Rectangular ROIs were placed directly adjacent to the RIBEYE immunosignals using the ROI manager of NIH ImageJ. Identical ROIs were used for retinal sections from CFA- and MOG/CFA-injected mice. For manually counting the number of immunolabelled puncta, the multipoint tool of NIH ImageJ was used. The number of puncta was related to the actual length of the scan areas that was determined using the measurement tool of NIS Elements AR 3.2, 64-bit. The average number of immunolabelled puncta was extrapolated for 60 μm length of OPL. For fluorescence intensity measurements, averages of the values were calculated and plotted as relative values (in %) normalized to CFA values. Box-and-whiskers plots were generated with Origin Pro 2018 software. A normal distribution of the data was tested by the Shapiro–Wilk normality test. If data were normally distributed, statistical significance was determined with Student’s *t*-test; non-normally distributed data were analyzed with Mann–Whitney U-test. Differences were considered to be statistically different with *p* < 0.05. Statistical analyses were performed with Origin Pro 2018 software.

### 4.7. Super-Resolution Structured-Illumination-Microscopy (SR-SIM) and Quantitative Analyses of Immunosignals

SR-SIM was performed as previously described [24,25,37,105,125]. For SR-SIM, an ELYRA PS1 setup (Carl Zeiss Microscopy GmbH; Oberkochen, Germany) was used. Images were acquired with a 63×/1.4 NA oil (DIC) objective using the 561 nm laser line and collected through an Andor iXon EM-CCD camera [25,37,105,125]. Data acquisition was performed in a blinded manner with the experimenter not knowing the identity of the samples. For 3D SR-SIM [132], 1.5-μm-thin immunolabelled resin sections were used and processed as described in [25,68]. Z-stack images were acquired with an interval of 125 nm between the individual z-planes using the ZEN 2010 software (black edition). The entire thickness of the retinal section was scanned, and images were then processed for 3D SR-SIM. Sections were oversampled to avoid signal loss during 3D reconstruction. Z-stack images of a single cropped RIBEYE-labelled synaptic ribbon were iteratively scanned to ensure the complete coverage of the immunosignal before proceeding to create the 3D view. Maximum 2D projection images were generated from the 3D images of single, cropped synaptic ribbons, as previously described [25,68]. The contour length of the maximum 2D projections (in μm) was determined with the open polynomial line option tool of the ZEN 2012 software (Figure 1(A2)). Average values were calculated and plotted in Microsoft Excel. Box-and-whiskers plots were generated with Origin Pro 2018 software. Statistical analyses of the SR-SIM data were performed as described above for the quantitative analyses of confocal images.

### 4.8. Transmission Electron Microscopy of Synapses from the Mouse Retina

Processing of mouse retina samples for transmission electron microscopy (TEM) was performed largely as previously described [30]. The posterior eyecups containing the retina were dissected from the isolated eyes within 5 min post-mortem and fixed first with 4% PFA (w/v) in PBS and next with 2.5% glutaraldehyde (v/v) in PBS (12 h each at 4 °C). Samples were osmicated with 1% OsO_4_ in 100 mM cacodylate buffer (1 h, 4 °C), contrasted with 2% uranyl acetate in 50 mM Na^+^-maleate buffer (pH 5.0) for 3 h at 4 °C. Samples were dehydrated in an ascending concentration series of ethanol and were finally equilibrated with propylene oxide (each step for 20 min at room temperature). Propylene oxide was gradually replaced by Epon/propylene oxide mixtures, as described [30]. Samples were infiltrated two times with pure Epon resin (≈10 h each) before polymerization at 60 °C for ≈36 h. Ultrathin sections (≈70 nm in thickness) were sectioned with an UltraCut E ultra-microtome (Reichert-Jung). The thickness of the ultrathin sections was estimated from the interference color of the ultrathin sections after floating off on water [133,134]. Images were acquired with transmission electron microscope (TEM (Tecnai 12 Biotwin; FEI, Eindhoven, The Netherlands) equipped with a Megaview III digital camera (Gatan) and controlled by iTEM acquisition software (Olympus, Hamburg, Germany). The TEM microscope was operated at 100 kV.

Photoreceptor synapses are highly enriched in the outer plexiform layer (OPL) and can be readily identified by TEM. The majority of photoreceptor synapses (>95% of total photoreceptor synapses in the mouse retina) are formed by rod photoreceptors [28,135,136]. Rod synapses can be readily identified in the OPL because of their typical appearance. Rod synapses possess a single large active zone with a single large ribbon. The presynaptic active zone is opposed by a typical, characteristic assembly of postsynaptic dendrites from horizontal- and bipolar cells.

### 4.9. Analysis of Synaptic Ribbon Height, Number of Docked Synaptic Vesicles, Ribbon-Tethered Synaptic Vesicles and Reserve Pool Synaptic Vesicles in Rod Photoreceptor Synapses of CFA-Injected Control Mice and MOG/CFA-Injected EAE Mice by Transmission Electron Microscopy

Quantitative analyses were performed on TEM images acquired from CFA-injected control mice and MOG/CFA-injected EAE mice on day 9 after injection. For measurement of synaptic ribbon height (defined as summarized in Figure 1(A1)), only rod photoreceptor synapses were analyzed in which the active zone could be clearly visualized and in which the typical postsynaptic configuration consisting of horizontal and bipolar cells was visible (postsynaptic triad/tetrad). By this way, only synaptic ribbons that were perpendicularly cut to the active zone were considered for the analyses. Photoreceptor synapses with anchored synaptic ribbon extending vertically from the active zone plasma membrane into the cytoplasm were considered for measuring the height of the ribbon. For the analyses of ribbon height in rod photoreceptor synapses electron micrographs of rod photoreceptor ribbon synapses were captured at a magnification of 48,000× for both CFA- and MOG/CFA-injected mice. Synaptic ribbon length was measured using the freehand line tool of NIH ImageJ, as indicated by the dashed orange line in Figure 4. The length of the scale bar that was present on the exported TEM images was used as reference for length calibration. Values were analyzed in Microsoft Excel, exported and plotted in Origin as bar graph and box-whisker plot. Shapiro–Wilk test was applied to determine whether data were normally or non-normally distributed. Since data were non-normally distributed, statistical significance was determined by non-parametric Mann–Whitney U-test.

TEM images at 48,000× magnification were also used to count the number of docked vesicles and synaptic vesicles tethered to the synaptic ribbon and for determining the synaptic vesicle density in cytosol (Figure 6). The number of vesicles tethered to the synaptic ribbon and the density of vesicle in the cytosol away from the synaptic terminal within a defined area (200 × 200 nm^2^) were counted manually using the multi point tool in Fiji software (NIH ImageJ). The number of docked synaptic vesicles at the active zone within 150 nm vicinity were counted from the TEM images of rod photoreceptor synapse in CFA control and MOG/CFA immunized mice, as previously described in [30] (Figure 6). NIH ImageJ software with multi point tool was used for counting the docked vesicles. Average number of docked vesicles from three mice each from CFA control and MOG/CFA immunized mice was plotted and statistical significance was determined by using Mann–Whitney U-test after applying the Shapiro–Wilk normality test that showed a non-normal distribution of data.

### 4.10. Estimation of Synaptic Ribbon z-Length in EAE- and Control-Injected Mice by TEM Analyses of Serial Ultrathin Sections

The length of rod synaptic ribbon in z-direction (Figure 1(A2) and Figure 5B), i.e., the long axis of the ribbon that runs in parallel to the active zone, was analyzed in serial ultrathin sections of rod photoreceptor synapses from MOG/CFA-injected EAE mice and CFA-injected control mice on day 9 after injection in the pre-clinical phase. Serial ultrathin sections were made from retina samples embedded for TEM as described above and collected on Formvar-coated slot grids. Up to 12–15 serial ultrathin sections were collected in the sequence of sectioning on a single slot grid (Figure 5A,B). Each grid was marked with a dot at one end to identify the first ultrathin section of the row. Images were acquired with transmission electron microscope (TEM) (Tecnai 12 Biotwin; FEI, Eindhoven, The Netherlands) equipped with a Megaview III digital camera (Gatan) and controlled by iTEM acquisition software (Olympus, Hamburg, Germany). The TEM microscope was operated at 100 kV. Transmission electron micrograph of rod photoreceptor ribbon synapse in the outer plexiform layer were acquired at magnification of 9900× to measure the synaptic ribbon length in Z-axis from the ultrathin serial section. Blood vessels and horizontal cell processes were used as a landmark to identify the same rod photoreceptor ribbon synapse in the OPL of the ultrathin sections.

The z-dimension of the synaptic ribbons in rod photoreceptor terminals was estimated by the number of serial sections in which an individual synaptic ribbon could be clearly identified. The number of sections per ribbon synapse were multiplied by 70 (nanometer thickness of one section) to obtain the Z-axis length of one synaptic ribbon (Figure 5B). A total of 317 (CFA) and 387 (MOG/CFA) rod photoreceptor ribbon synapses from CFA-injected control and MOG/CFA-injected EAE mice (obtained at day 9 after injection) were analyzed. Statistical analysis was performed by using Origin Pro 2018 software. The distribution of the data was checked by Shapiro–Wilk normality test. Non-parametric Mann–Whitney U-test was applied to determine the statistical significance. *p* < 0.05 was considered as statistically significant difference between the groups.

### 4.11. Preparation of Retinal Slices from MOG/CFA-Injected Transgenic SypHy Reporter Mice for the Analysis of Vesicle Cycling in Rod Photoreceptor Synapses

Preparation of retinal slices was performed as previously described [24]. MOG/CFA- and CFA-injected SypHy reporter mice were sacrificed on day 7, day 8, or day 9 after injection and their retinas were isolated within 5 min post-mortem. Briefly, enucleated eyes were punctured at the equatorial plane with a 20 G syringe needle and bisected at the equatorial plane. The posterior eye cup was transferred into LCS buffer (RT). LCS was saturated with 5% CO_2_, 95% O_2_ before use. From the posterior eye cup, the retina was gently peeled off. Four cuts were made in the retina so that it could be flat-mounted onto black-gridded nitrocellulose filter membranes (Millipore; Darmstadt, Germany; #HABG01300) with ganglion cell side facing nitrocellulose membrane. Membrane filters with the attached retina and some LCS solution (to prevent drying of the retinas) were transferred to a silica sieve funnel and gentle suction was applied to enhance adhesion of the retina to the filter membrane via the attached syringe.

The nitrocellulose filter with the attached retina on top was transferred to a glass slide, some streaks of Vaseline were applied to the glass slide to prevent lateral movements of the filter during subsequent slicing. The glass slide with the retina attached on nitrocellulose filter was transferred to the cutting stage of Werblin-type tissue slicer. Retina slices of ≈200 µm thickness were sectioned with the slicer. Slices were then immediately transferred onto a glass coverslip. The gaps between the streaks of Vaseline were filled with LCS solution. LCS was saturated with 5% CO_2_, 95% O_2_ before use. Slices were carefully picked from the cutting platform with fine tweezers, turned vertically so that all retinal layers were visible from the photoreceptors on the free outer side of the slice to the ganglion cells that were facing toward the filter surface, and fixed between the Vaseline streaks.

### 4.12. Optical Recording of Synaptic Activity in MOG/CFA-Injected SypHy Reporter Mice

Optical recording experiments were performed as previously described [24] with female 10–12 weeks old SypHy transgenic reporter mice of C57BL/6J genetic background. Retinal slices (≈200 µm thick), prepared from CFA- or MOG/CFA-injected transgenic SypHy reporter mice on day 7, 8 or 9 after injection, were incubated in the dark at 29 °C temperature for 15 min in LCS in a 5% CO_2_ incubator. The coverslip containing the retina slice was placed in the holding chamber and rinsed three times with 2 mL RS solution gassed with 5% CO_2_, 95% O_2_. After washing, the holding chamber was filled with RS to submerge the slice and was then transferred to the recording chamber of the A1R Confocal microscope (Nikon) for fluorescence imaging. Recording chamber temperature was maintained at 28 °C by a temperature controller (Harvard Instruments) throughout the recording experiment. A Nikon plan Fluor 10 × 0.3 W DIC N1 water immersion objective lens was used for fluorescence imaging.

For the optical recording of exo-/endocytosis at photoreceptor synapses, at first one minute of baseline fluorescence was recorded in gassed RS solution (saturated with 5% CO_2_, 95% O_2_) followed by depolarization with 25 mM KCl-containing RS solution (denoted as DS, depolarization solution) for one minute to elicit exocytosis. After depolarization with DS solution, the slices were repolarized for one minute by adding 2 mL RS solution (saturated with 5% CO_2_, 95% O_2_) to the chamber for vesicle retrieval and to return to baseline fluorescence as the vesicles re-acidify. Each slice was stepped through three depolarization-recovery cycles (by adding DS solution followed by its replacement with RS solution). The depolarizing and recovery solutions (DS and RS, respectively, 2.0 mL each) were added manually to the holding chamber from one side using a dropper and the other side suction was applied to remove the solutions. The responses were measured by making a rectangular region of interest after focusing on the photoreceptor synapse layer (OPL) with an acquisition rate of 1 Hz at emission wavelength of 545 nm and excitation wavelength of 488 nm.

### 4.13. Data Analyses of SypHy Responses

Data were exported from NIS Elements software to Excel for further analysis and normalized by setting the fluorescence baseline to one arbitrary unit (A.U.). Curve fitting was performed with Igor Pro software in order to measure amplitudes of depolarization and recovery responses from each slice. A double exponential curve was fitting best for both the depolarization and repolarization responses demonstrating fast and slow mechanisms of vesicle cycling in rod photoreceptor synapses. The resulting values for amplitudes were averaged and compared for statistical significance between the MOG/CFA-injected EAE mice and CFA-injected control mice. Statistical analysis was performed using Origin pro 2018 software and GraphPad 8 software. Sample with a normal distribution (Shapiro–Wilk test) were compared using unpaired Student’s *t*-test. Mann–Whitney, a non-parametric test, was applied for comparing samples which were not normally distributed according to Shapiro–Wilk test. Kruskal–Wallis ANOVA with Dunn post-hoc analysis was performed for multiple comparison of non-normally distributed data.

### 4.14. Measurement of Endocytosis at Photoreceptor Synapse of MOG/CFA-Injected EAE Mice and CFA-Injected Control Mice Using the Vesicular Proton Pump Inhibitor Bafilomycin A1

As described above, fusion of SypHy-containing, acidic synaptic vesicles with the presynaptic plasma membrane leads to an increase of SypHy fluorescence that is followed by a decrease in SypHy fluorescence caused by vesicle endocytosis and re-acidification. Vesicle endocytosis is likely accompanied by ongoing vesicle exocytosis in the continuously active photoreceptor ribbon synapses. In order to more precisely compare endocytosis at photoreceptor synapses of MOG/CFA-injected EAE mice in comparison to CFA-injected control mice, we used bafilomycin, bafilomycin A1, a reversible inhibitor of the vesicular V-type ATPase proton pump [137]. By this way, bafilomycin blocks the re-acidification of endocytosed vesicles and the quenching of SypHy fluorescence [138,139,140]. Optical recording of endocytosis in the presence/absence of bafilomycin A1 was determined as previously described [138,141]. To obtain values for endocytosis, fluorescence in the presence of bafilomycin A1 [+baf] was subtracted from the fluorescence in the absence of bafilomycin A1 [−baf], i.e., [−baf]-[+baf]. The inhibition of the vesicular V-type ATPase proton pump by bafilomycin A1 is a frequently used and well-established approach to measure endocytosis in synapses [137,138,139,140,141,142,143]. Recently, also additional effects of bafilomycin A1 (e.g., on membrane fusion/autophagy/mitochondrial function) have been observed that mostly result from its inhibitory effect on proton pumps [143,144,145]. Additional effects could also apply [145,146].

All optical recordings were done with retinal slices obtained from CFA- and MOG/CFA-injected SypHy reporter mice at day 9 after injection. Quantitative measurements of fluorescence signals at individual retina slices were performed by making a region of interest (ROI) around the OPL with NIS Elements ROI manager, as previously described [24,25]. The SypHy responses were recorded with an acquisition rate of 1 Hz using the 488 nm laser excitation line and recording fluorescence emission at 545 nm.

### 4.15. Measurement of Basal (“Resting”) Endocytosis at Photoreceptor Synapse of MOG/CFA-Injected EAE Mice and CFA-Injected Control Mice Using Bafilomycin A1

For the measurement of basal endocytosis, optical recording was performed during incubation with RS (“resting phase”) in retinal slices from CFA-injected control and MOG/CFA-injected EAE SypHy transgenic mice obtained on day 9 after injection. The retinal slices were pre-incubated with or without bafilomycin A1 for 15 min before optical recording. The basal fluorescence of CFA- or MOG/CFA slices was recorded for 1 min in RS with or without bafilomycin A1. SypHy fluorescence in the absence of bafilomycin A1 provides a readout of vesicle exocytosis diminished by the drop of SypHy fluorescence that results from vesicle endocytosis and subsequent quenching of SypHy fluorescence by re-acidification of the endocytosed vesicles ([exocytosis]–[endocytosis]). SypHy fluorescence signals in the presence of bafilomycin A1 reflect a readout of exocytosis only because re-acidification of endocytosed vesicles (and thus SypHy fluorescence quenching by acidic pH) is blocked by bafilomycin and thus does not lead to quenching of SypHy fluorescence [138,141,142]. To obtain values for endocytosis during RS incubation (resting phase), the basal fluorescence response in the presence of bafilomycin A1 [+baf] was subtracted from the basal fluorescence response in the absence of bafilomycin A1 [−baf], i.e., [−baf]-[+baf], during incubation with RS.

### 4.16. Measurement of Post-Stimulus Endocytosis at Photoreceptor Synapse of MOG/CFA-Injected EAE Mice and CFA-Injected Control Mice Using Bafilomycin A1

In the post-stimulus recovery phase, endocytosis and subsequent vesicle re-acidification resulted in the return of SypHy fluorescence to resting levels (Figure 7). Post-stimulus vesicle endocytosis likely occurs together with vesicle exocytosis at the continuously active photoreceptor ribbon synapses. Thus, we performed optical recordings in the presence/absence of bafilomycin A1 during the post-stimulus recovery phase in order to more precisely determine endocytosis in photoreceptor synapses of MOG/CFA-injected EAE mice in comparison to CFA-injected control mice in that phase. Bafilomycin A1 (5 µM final concentration in depolarization solution containing 0.1% DMSO; 0.1% DMSO without bafilomycin A1 as control) was added to these experiments at the beginning of the 60-sec-lasting depolarization to make sure that the slices were equilibrated with bafilomycin A1 in the post-stimulus recovery phase. After depolarization, RS with or without bafilomycin (as indicated in the respective experiment) was added to measure vesicle endocytosis, as described above.

### 4.17. Bafilomycin Data Analysis

SypHy fluorescence values from CFA-injected control and MOG/CFA-injected EAE mice day 9 post injection were recorded in the presence or absence of bafilomycin and analyzed with Microsoft Excel. The change in fluorescence was normalized to the baseline resting fluorescence, if not indicated otherwise. The normalized fluorescence in the presence and absence of bafilomycin was used to obtain the total endocytosis. Total endocytosis (F_endo_) was obtained by subtracting the fluorescence values in the presence of bafilomycin (F_SypHy+baf_) from the fluorescence values in the absence of bafilomycin (F_SypHy-baf_). [F_endo_ = F_SypHy-baf_ − F_SypHy+baf_], as described by [138]. The double exponential curve fit analysis was performed using Igor Pro software to obtain the amplitudes of endocytosis in CFA control and MOG/CFA immunized mice.

### 4.18. Western Blot Analyses

For quantitative immunoblotting, retinas from CFA and MOG/CFA mice were isolated within 5 min post-mortem. Retinas were lysed in 1% Triton X-100, 20 mM Tris pH-8.00, 200 mM NaCl, 1mM DTT, complete EDTA-free protease inhibitor cocktail (Roche) for 15 min on ice. Protease and phosphatase inhibitor cocktail (PPC1010-sigma) was used to prepare the retina lysate for immunoblotting with p-dynamin 1 (Ser-774) antibody. Retinas in the lysis buffer were sonicated twice for 1 s at 10% power using ultrasonic probe (Bandelin Sonoplus). After sonication lysate was kept in ice for 15 min and followed by centrifugation at 10,000× *g* for 10 min at 4 °C. Supernatant was collected and protein concentration was determined by the Amido Black method as described by [147]. Equal protein amount (30 µg) of the retinal lysates from CFA control and MOG/CFA immunized mice retina was separated by 10% SDS-PAGE followed by wet transfer on to nitrocellulose membrane in the cold room at 50 V for 5 h. The next day, the membrane was blocked with 5% skim milk for 1 h at RT to avoid non-specific binding of the primary antibody. Membrane was incubated with the primary antibody in 5% skim milk overnight, at 4 °C. The following day, after several washes with PBS to remove unbound primary antibody, membrane was incubated with the respective IRDye secondary antibodies (LI-COR) at a dilution of 1: 5000. Antibody signals were detected by an Odyssey Imager (LI-COR). The band intensities were quantified by using densitometry in Image Studio Lite software (LI-COR). The band density of protein of interest was normalized to the band density of loading control protein actin in the same lane. Student’s *t*-test was applied for determining the statistical significance between the CFA and MOG/CFA mice. The number of mice used from CFA-control and MOG/CFA immunized mice are indicated in the respective figures.

### 4.19. Dot Blot Analyses of Anti-p-Dynamin1 Antibody on p-Dynamin1 and p-Dynamin3 Peptides Cross Linked to Bovine Serum Albumin (BSA)

The anti-p-dynamin1 (Ser774) antibody was tested by dot blot analyses on p-dynamin1 and p-dynamin3 peptides that were cross-linked to bovine serum albumin (BSA), as previously described [55]. The following dynamin peptides were used for dot blot analyses: p-dynamin1: DDSWLQVQSVPAGRR**S**PTSSPTPQR (serine underlined in the peptide sequence has been phosphorylated); p-dynamin3: DDSWLQHSRR**S**PPPSPTTQR (serine underlined in the peptide sequence has been phosphorylated). Phospho-peptides were obtained from Pepmic (Suzhou, China). For coupling, 50 µg of each of the peptides were incubated with 50 µg of BSA in a total volume of 100 µL in the presence of 1% glutaraldehyde in PBS for 1 h on ice. After 1 h, 0.1% NaBH4 was added to the reaction mixture and kept at RT for 15 min to block the free unreacted aldehyde groups. After 15 min, peptides were spotted on nitrocellulose membrane in a volume of 10 µL. The total amount of the peptides spotted is given in Figure 15. Equal concentration of BSA only and an unrelated control peptide (CENEIQDLLRAKRELESKLQRLQAQG) were also spotted that served as negative controls to test for unspecific binding of antibodies. Approximately 20 min after spotting of the peptides, the dried membrane with peptides was stained with Ponceau-S and documented with a GelDoc system and Quantity One Software (BioRad; Feldkirchen, Germany) using epi-white illumination. After de-staining with PBS, the blotting membrane was incubated with 5% skimmed milk in PBS for 1 h at RT to block unspecific protein-binding sites. After blocking, the membrane was incubated with primary antibody (p-dynamin1 (Ser774), diluted 1:1000 in 1% skimmed milk in PBS) for overnight in cold room. After overnight incubation, the membrane was washed with PBS and incubated with the respective secondary antibodies (rabbit anti-sheep conjugated to HRP; diluted 1:3000 in 1% skimmed milk for 2 h at RT). Binding of the antibodies was detected by enhanced chemiluminescence, as previously described [24,25,55].

## Figures and Tables

**Table 1:** Primary antibodies.

Antibody	Reference	Dilution
Anti-RIBEYE(B), rabbit polyclonal (U2656)	[29]	1:1500 (IF)
Anti-RIBEYE(B), mouse monoclonal (2D9)	[24]	1:1000 (IF)
Anti-Dynamin1xb (mouse monoclonal)	[55]	1:200 (IF) 1:500 (WB)
Anti-Dynamin3 (rabbit polyclonal)	Synaptic Systems, (Göttingen, Germany) SYSY-115302 [52]	1:500 (IF) 1:1000(WB)
Anti-phospho-Dynamin1 (Ser774; sheep polyclonal)	Santa Cruz, Sc-135689 (Heidelberg, Germany) [126]	1:100 (IF) 1:500 (WB)
Anti-Actin, mouse monoclonal (clone C4)	Millipore, MAB1501 (Darmstadt, Germany) [55]	1:1000(IF) 1:2000(WB)

**Table 2:** Secondary antibodies.

Antibody	Source	Dilution
Chicken anti-mouse Alexa488	Invitrogen Molecular Probes (Karlsruhe, Deutschland), A-21200	1:1000 (IF)
Donkey anti-rabbit Alexa568	Invitrogen Molecular Probes (Karlsruhe, Deutschland), A-10042	1:1000 (IF)
Chicken anti-rabbit Alexa488	Invitrogen Molecular Probes (Karlsruhe, Deutschland), A-21441	1:1000 (IF)
Donkey anti-mouse Alexa568	Invitrogen Molecular Probes (Karlsruhe, Deutschland), A-10037	1:1000 (IF)
Donkey anti-sheep Alexa 568	Invitrogen, Molecular Probes (Karlsruhe, Deutschland), A-21099	1:1000 (IF)
Donkey anti-rabbit Alexa 647	Invitrogen Molecular Probes (Karlsruhe, Deutschland), A-31573	1:1000 (IF)
Goat anti-rabbit peroxidase-conjugated (POX)	Sigma (Taufkirchen, Germany), A-6154	1:3000 (WB)
Rabbit anti-sheep HRP-conjugated	Invitrogen ThermoFisher (Langenselbold, Germany), 31480	1:3000 (WB)
Goat anti-mouse POX	Sigma (Taufkirchen, Germany), A-3673	1:3000 (WB)
IRDye^®^ 680LT Donkey anti-Mouse IgG	LI-COR (Bad Homburg, Germany), 92568022	1:5000 (WB)
IRDye^®^ 800CW Donkey anti-Rabbit IgG	LI-COR (Bad Homburg, Germany), 92532213	1:5000 (WB)
IRDye^®^ 800CW Donkey anti-Goat IgG	LI-COR (Bad Homburg, Germany), 92832214	1:5000 (WB)

## Data Availability

Available upon request.
